# Inference of a three-gene network underpinning epidermal stem cell development in *Caenorhabditis elegans*

**DOI:** 10.1016/j.isci.2025.111826

**Published:** 2025-01-16

**Authors:** Alicja Brożek, Arianna Ceccarelli, Andreas Christ Sølvsten Jørgensen, Mark Hintze, Vahid Shahrezaei, Michalis Barkoulas

**Affiliations:** 1 Department of Life Sciences, Imperial College London, London SW7 2AZ, UK; 2 Department of Mathematics, Imperial College London, London SW7 2BX, UK; 3 Mathematical Institute, University of Oxford, Oxford OX2 6GG, UK; 4 I-X Centre for AI In Science, Imperial College London, London W12 0BZ, UK

**Keywords:** Bioinformatics, Biological sciences, Cell biology, Gene network

## Abstract

Gene regulatory networks are crucial in cellular decision-making, making the inference of their architecture essential for understanding organismal development. The gene network of *Caenorhabditis elegans* epidermal stem cells, known as seam cells, remains undefined. Here, we integrate experimental data, mathematical modeling, and statistical inference to investigate this network, focusing on three core transcription factors (TFs), namely ELT-1, EGL-18, and CEH-16. We use single-molecule FISH to quantify TF mRNA levels in single seam cells of wild-type and mutant backgrounds across four early larval stages. Using Modular Response Analysis, we predict TF interactions and uncover a repressive interaction between CEH-16 and *egl-18* consistent across time points. We validate its significance at the L1 stage with ordinary differential equations and Bayesian modeling, making testable predictions for a double mutant. Our findings reveal TF regulatory relationships in seam cells and demonstrate a flexible mathematical framework for inferring gene regulatory networks from gene expression data.

## Highlights

• We construct a quantitative gene network for seam cells from gene expression data
• We find that CEH-16 represses *egl-18* throughout early larval stages
• Mathematical modeling and inference validate the importance of this interaction
• Our model accurately predicts mRNA levels upon the perturbation of *ceh-16* and *egl-18*

## Graphical abstract

**Figure:**
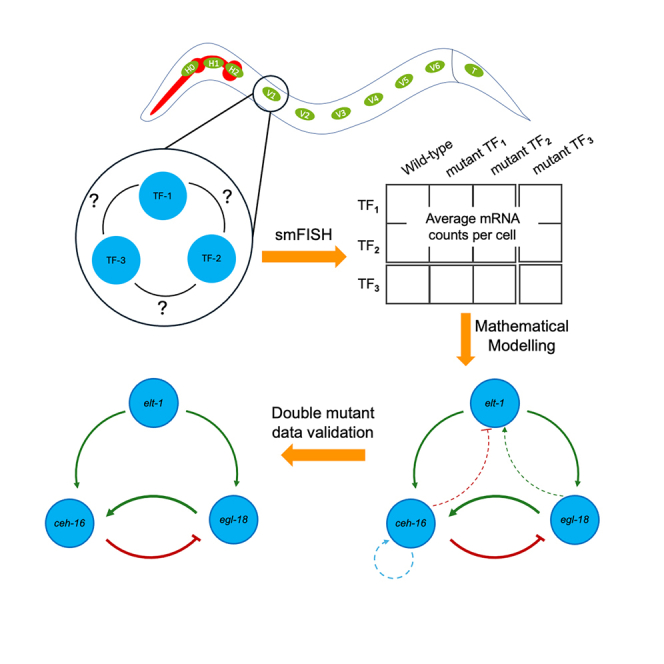

## Introduction

**Figure 1:**
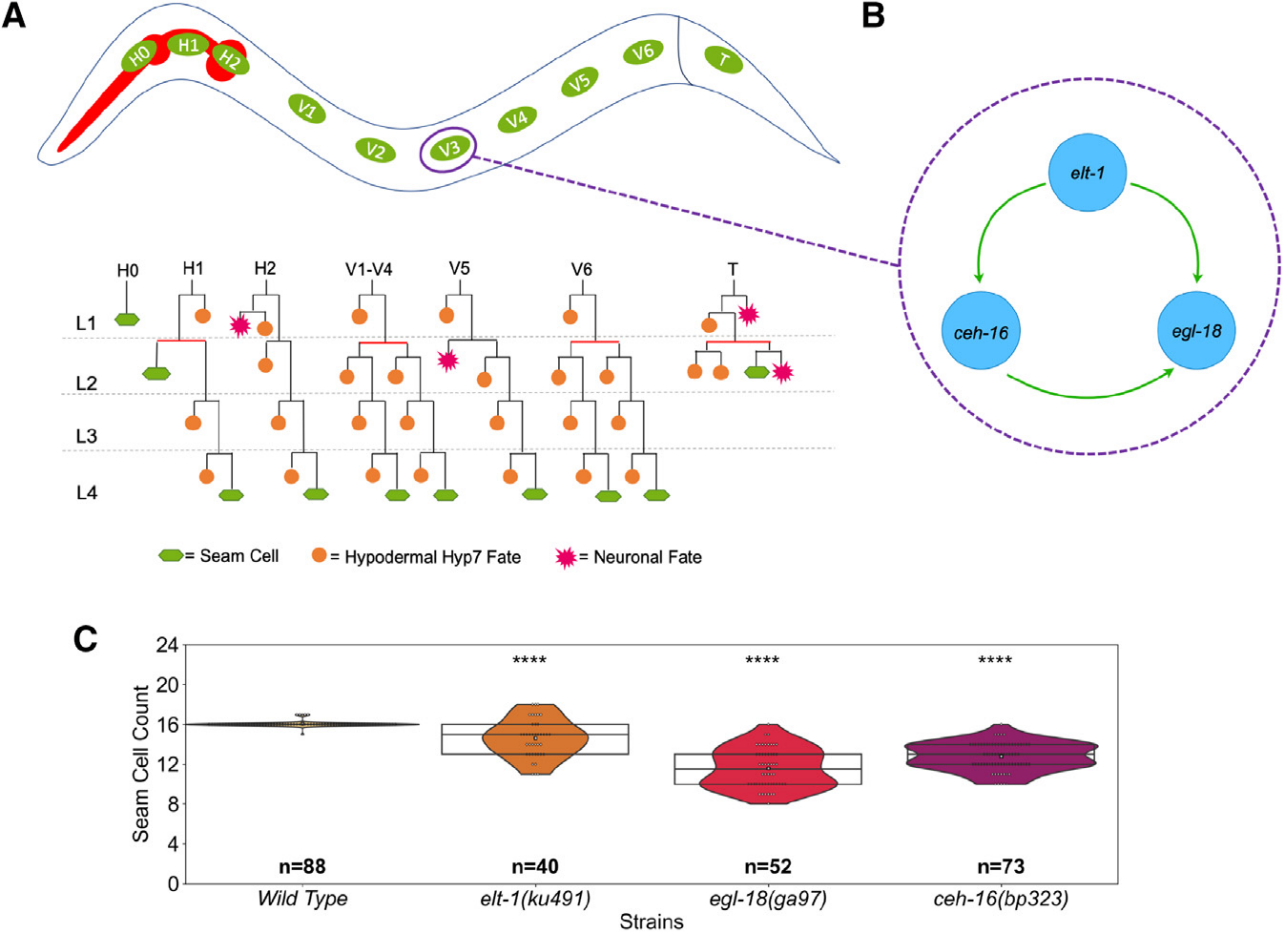
The core seam cell and the core TF seam cell gene network (A) An illustration of the stereotypic division patterns of seam cells. Seam cell fate is denoted in green, hypodermal cell fate is denoted in blue, and neuronal fate is shown in magenta. Red bars indicate symmetric divisions. (B) A schematic of the gene network compiled from interactions proposed in previous literature: ELT-1 activates both *ceh-16* and *egl-18* expression, and CEH-16 activates *egl-18* expression. (C) Seam cell number comparison at the end of the L4 larval stage between the wild type and animals carrying mutations in core seam cell TFs. The lines of the box represent the quartiles of the data, and the white star in the middle represents the median. The *p*-values are calculated using the Z-test and are shown as ∗ = *p <* 0.05, ∗∗ = *p <* 0.01, ∗∗∗ = *p <* 0.001, ∗∗∗∗ = *p <* 0.0001. The *n* value on the graph corresponds to the number of animals counted.

Describing mechanisms of stem cell patterning is fundamental in increasing our understanding of tissue homeostasis. The model nematode *Caenorhabditis elegans* is well known for its robust development with high consistency in cell numbers and types of cells produced. A key cell population in *C. elegans* that exhibits stem cell-like behavior and generates the majority of its epidermis are the so-called seam cells. Over the four larval stages of *C. elegans* post-embryonic development, the seam cells divide at regular time points once or twice per larval stage (Figure 1A). The vast majority of their divisions are asymmetric and produce one seam cell and one hypodermal cell or neuroblast.^1^ *C. elegans* hatches with 10 cells per lateral side, but during the L2 larval division, several seam cells also divide symmetrically to increase their population from 10 to 16.^1^^,^^2^ It is largely unknown how seam cells make robust choices between undergoing self-renewal or differentiating and how this relates to cell lineage choices across developmental time points.^3^^,^^4^ Given the stem cell behavior and experimental tractability of the system, the epidermis of *C. elegans* provides an invertebrate model to dissect the mechanisms underlying stem cell regulation and developmental robustness.

Genetic analysis has revealed a number of genes that are of paramount importance to the regulation of seam cell behavior. ELT-1 is a GATA factor whose function is linked to seam cell fate specification and maintenance. *elt1* is expressed in the whole epidermis in the embryo and then localizes to the seam cells post-embryonically.^5^^,^^6^^,^^7^ Complete knockouts of *elt-1* are lethal because they are unable to specify an epidermis, but hypomorphic mutations that partially reduce gene function such as *elt-1(ku491)* result in a reduced terminal seam cell number. ELT-1 regulates other seam-cell-specifying TFs such as BRO-1, which is part of the BRO-1/RNT-1 (CBFβ/RUNX) complex that influences the choice of cells to divide symmetrically.^8^ CEH-16 is a homolog of the Drosophila gene *engrailed* as well as the human En1 and En2.^9^ It is expressed in the AB lineage and plays a role in embryonic seam cell development.^10^ During post-embryonic development, *ceh-16* is specifically expressed in the seam cells where it is required for the L2 symmetric seam cell division.^11^ The complete knockout of *ceh-16* is embryonic lethal, a *ceh-16(bp323)* hypomorphic allele results in a seam cell loss, while *ceh-16* overexpression results in seam cell hyperplasia through the conversion of normally asymmetric divisions to symmetric.^11^ EGL-18 is another GATA factor that is thought to act downstream of the Wnt signaling pathway.^12^^,^^13^ EGL-18 is thought to promote seam cell fate in posterior seam cells following asymmetric division partly by repressing the differentiation program, a function which is shared with CEH-16.^10^^,^^13^ Strong *egl-18* loss-of-function mutations are not lethal possibly due to the compensation by the paralog GATA factor ELT-6.^14^ Nevertheless, in the absence of *egl-18*, a number of cells lose their ability to maintain the seam cell fate.^13^^,^^15^ While there is some evidence to suggest that *elt-1*, *egl-18,* and *ceh-16* may interact,^10^^,^^16^^,^^17^ the exact architecture of their interaction network has so far not been elucidated. The literature portrays ELT-1 as an early specification factor promoting the expression of both *ceh-16* and *egl-18*, while CEH-16 promotes *egl-18* expression (Figure 1B).^16^ Understanding the way these core TFs interact is essential for understanding the patterning rules for this stem cell population.

Inference methods for gene regulatory networks (GRNs) are dictated by the type of data that is available and the type of network abstraction or GRN model that one wishes to assemble.^18^^,^^19^^,^^20^^,^^21^^,^^22^^,^^23^ The data used could include protein-DNA, protein-protein interaction data or gene expression data at the bulk or single-cell level. One of the simplest levels of abstraction for a GRN model is a static graph, capturing gene-gene regulatory or correlational links. An advantage of static models is that they contain fewer parameters; thus, their inference scales well computationally to larger networks. In order to tell apart correlation from causal relationships, perturbation-based data such as gene expression in genetic mutants could be used. One method to develop a directed graph model for a GRN using such data, with a quantitative measure of interaction strength, is the Modular Response Analysis (MRA).^24^ MRA allows for the construction of a quantitative static network using expression data from perturbation experiments without requiring detailed mechanistic information on the chemical reactions involved.

In contrast to static models, dynamic GRN models can include mechanistic information and are more useful in producing predictions. These include logical or Boolean models, ordinary differential equation (ODE) models, and stochastic discrete models simulated using the Gillespie algorithm.^18^^,^^19^^,^^20^ Dynamic GRNs typically have many unknown parameters and thus are harder to infer, particularly for larger networks. The task is to infer both the architecture and the parameters of such GRN models from available data. This kind of inference problem is sometimes called equation or model discovery.^25^^,^^26^^,^^27^ A Bayesian model discovery approach called SLING (Sparse Likelihood-free Inference using Gibbs) uses sparsity-inducing priors to find the simplest model among a library of related models that can explain a given set of data.^28^ This approach, which is simulation-based and related to Approximate Bayesian Computation (ABC),^29^ is applicable to non-linear or stochastic models of biochemical networks. The method has been used for the inference of GRNs based on synthetic data,^28^ but it remains to be applied to real data.

In this study, we use single-cell snapshot transcription data combined with mathematical and statistical analysis to systematically infer a core TF GRN for the seam cells at 4 different time points during early post-embryonic development in *C. elegans*. To understand how disruption in the activity of each core TF might affect the expression pattern of other genes within the network, we use single-molecule Fluorescent *In Situ* Hybridization (smFISH),^30^ which provides information on mRNA expression at the single cell level in wild type and loss-of-function mutants. Next, we use a modified version of the MRA algorithm to test links between components in the network and estimate the coefficients of their interaction.^24^ We found that the most consistent interaction was the repression of *egl-18* by CEH-16. Given that this interaction opposed existing literature,^11^ we decided to further evaluate it mathematically. Based on the network layout at the late L1 time point, we constructed a detailed ODE model of the GRN and used the recently developed Bayesian model discovery tool SLING^28^ to both estimate the parameter values and assess which links are necessary for the gene network to explain and predict experimental data. This model allowed accurate predictions of the network upon the combined perturbation of *ceh-16* and *egl-18*. Taken together, we propose that the repressive interaction between CEH-16 and *egl-18* is a key feature of the seam cell network.

## Results

### Gene expression analysis of core seam cell transcription factors via single molecule fluorescent *in situ* hybridization in early larval development

**Figure 2:**
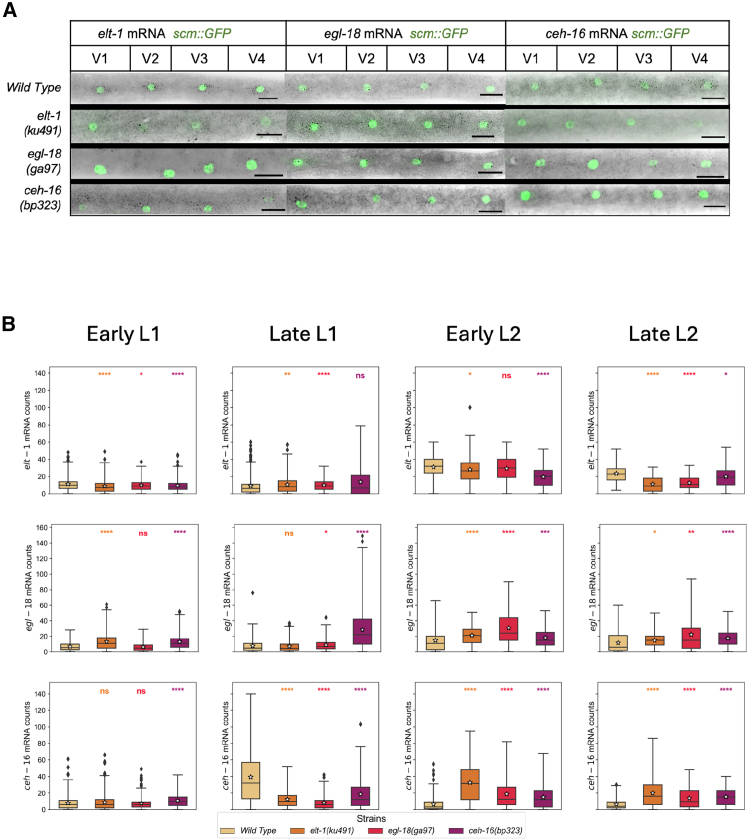
smFISH gene expression analysis in wild type and TF mutant backgrounds (A) Representative smFISH images in wild type and TF mutant backgrounds. The mRNAs are visualized as black spots, and the nucleus of the seam cells appears green because all animals carry a seam cell marker (scmGFP) in the background. The scale bar at the bottom right indicates a 10μm length. (B) Quantification of smFISH signal in V1-V4 seam cells where *n >* 60 cells were used for each quantification. The boxplots indicate the interquartile range (IQR) of the data, whiskers extend to the furthest data points within 1.5 times the IQR, the black line represents the median, and the white square the mean. The significance of the changes between mutant and wild-type backgrounds was calculated using the Mann-Whitney U test and is presented as follows: ∗ = *p <* 0.05, ∗∗ = *p <* 0.01, ∗∗∗ = *p <* 0.001, ∗∗∗∗ = *p <* 0.0001. Strains are color-coded as indicated in the key underneath the panel. See also Figures S1–S3.

To resolve the interrelationship between three core TFs of the seam cell gene network, namely ELT-1, CEH-16 and EGL-18, we compared gene expression between the wild type and corresponding loss-of-function mutants. Given that both *ceh-16* and *elt-1* are essential genes in *C. elegans*, we utilized hypomorphic mutations that are viable yet display a significant decrease in seam cell number at a comparable level to strong loss-of-function alleles of *egl-18*^31^ (Figure 1C). We used smFISH to quantify mRNA levels in individual seam cells across four time points in early larval development (Figures 2A and 2B). These time points were chosen to reflect the seam cell state before and after the L1 asymmetric division as well as the state following the L2 symmetric and L2 asymmetric division. We use here the approximate staging terms “early” or “late” L1/L2 respectively to refer to these four time points for simplicity. We focused primarily on V1-V4 lineages as these seam cells are in close proximity along the body axis and divide in a similar manner (Figures 1A and 2A). In the wild type, both *elt-1* and *egl-18* have the highest expression in early L2, and while *egl-18* expression remains fairly consistent during L1, *elt-1* expression is more variable (Figures 2B and S1). *ceh-16* expression peaks earlier, at early L1 and appears to shows high variability across the time points (Figures 2B and S1).

The impact of the mutations on core TF gene expression was dependent on the exact time point. For example, *ceh-16* levels were decreased in mutant backgrounds in late L1, however they were increased at L2. *elt-1* levels were decreased in *ceh-16(bp323)* background at early L1 and L2, but at late L1 they were unaffected. Both L2 time points showed some repression of *ceh-16* and *egl-18* by ELT-1. The most consistent difference between wild type and mutant backgrounds across stages was the change in *egl-18* expression in the *ceh-16(bp323)* mutant background. At every time point, we observed an increase in *egl-18* expression in the absence of *ceh-16*, indicating that CEH-16 might be an *egl-18* repressor. Therefore, we considered this repressive interaction to be robust and sought to test its importance for the network *egl-18* mathematically.

We note that expression for all core TF genes showed high levels of animal-to-animal variability characterized by Fano factors well above 1 (Figure S2A), which is the baseline variability for a Poisson process. The large variability can also be illustrated by the coefficient of variation (Figure S2B), which was between 0.6 and 1.2 for all genes and backgrounds. Fano factors above 1 can be indicative of bursty gene expression, but in this case we found high correlations of the expression of the same gene between two V1-V4 seam cells in the same animal, which indicates the presence of large animal-to-animal extrinsic variability, perhaps caused by extrinsic factors such as subtle differences in cell size or cell cycle (Figure S3). This extrinsic variability can mask the information contained in the intrinsic noise. Therefore, in this study, we chose to focus mainly on the effects of the perturbation on the mean expression.

### Constructing a gene regulatory network using modular response analysis

The smFISH findings challenged the network in the literature consisting of a positive action from CEH-16 on *egl-18* and from ELT-1 on *ceh-16* and *egl-18*.^16^ However, the results were also noisy and varied as a function of time in development and, as such, difficult to interpret without systematic analysis. Therefore, we used mathematical modeling and statistical inference to deduce the key features of the topology of the underlying GRN by characterizing the strength and importance of interactions. First, we carried out MRA to estimate the strengths of putative links between the genes of the network at different time points.^24^ The MRA algorithm was originally designed for cases where there is a change in the quantity of available protein, for example, upon RNAi knockdown. Here, we adapted the MRA algorithm for use in cases where protein activity is decreased without a change in protein levels, as expected in the case of hypomorphic alleles. The MRA produces an interaction matrix where the value of each column indicates the gene carrying out the action, the row indicates the gene receiving the action, and the cell at the overlap between the row and column indicates the strength of the interaction and whether it is activatory (positive) or repressive (negative).

**Figure 3:**
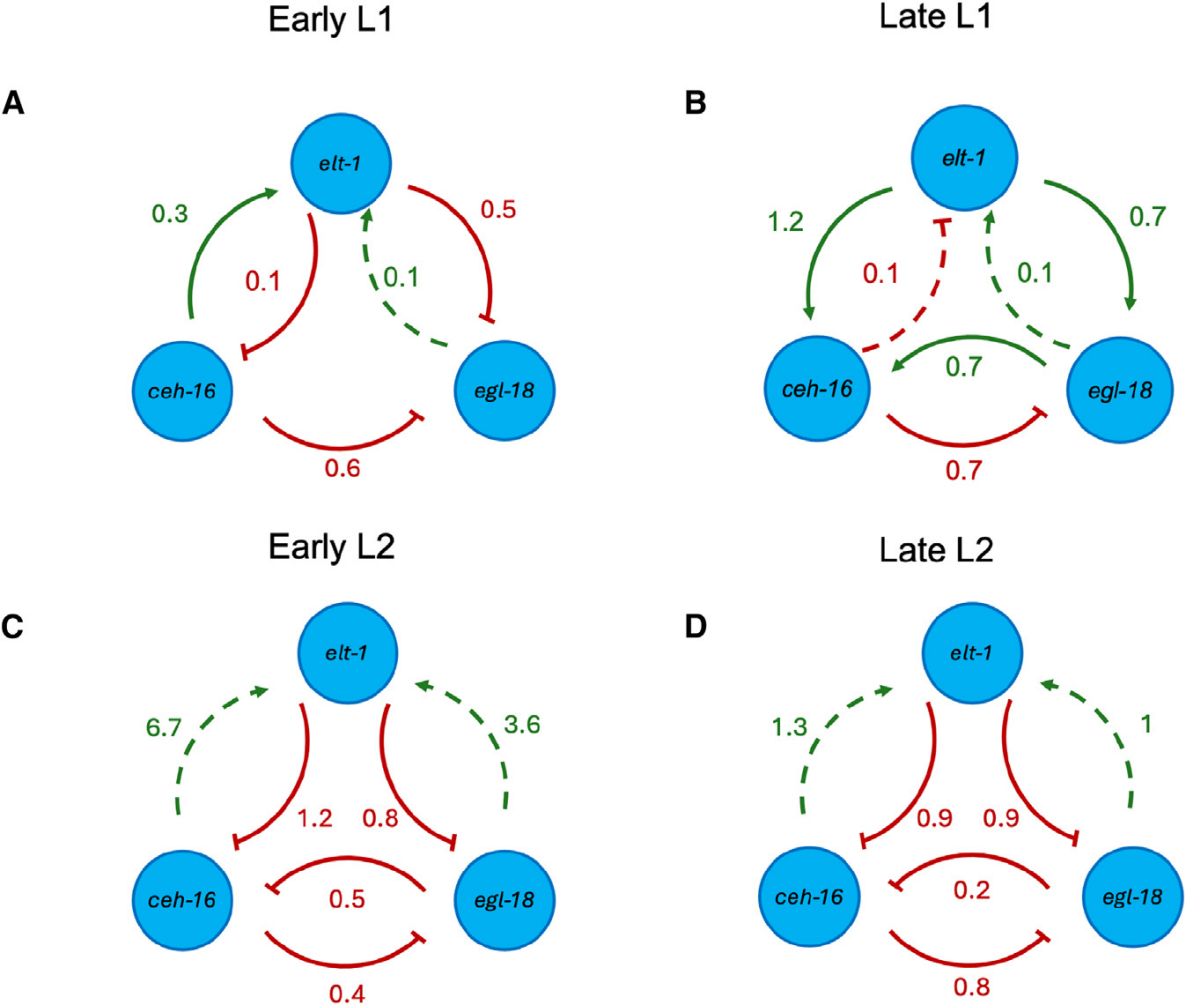
Modular Response Analysis (MRA) to construct a seam cell gene network (A–D) Network summarizing the results of the MRA algorithm using the average of each interaction for early L1 (before the first asymmetric division shown in A), late L1 (after the first asymmetric division in B), early L2 (after the L2 symmetric division in C) and late L2 (after the L2 asymmetric division in D). Green arrows represent activation, while orange/red blunt-end arrows represent repression. Dashed arrows show interactions where the error bars of the values crossed 0. See also Figures S4 and S5.

The MRA algorithm was applied to the smFISH data collected from V1-V4 cells, and using bootstrapping for 10000 iterations, the MRA sampled data with replacement to generate the interaction matrix (boxplots of the coefficients of the 10000 interaction matrices generated are shown in Figure S4). The resulting interaction was obtained by taking the average coefficient values, thereby generating summary GRNs at the four time points (Figures 3A–3D). Connections with an average coefficient value near 0 and error bars crossing 0 represent weakly supported connections and are denoted by a dashed line in (Figures 3A–3D). MRA identified several strong connections. In the early L1 stage, strong connections were the activation of *elt-1* by CEH-16 and repression of *egl-18* by both ELT-1 and CEH-16 (Figure 3A). At late L1, strong connections were the positive effect of ELT-1 on *egl-18* and *ceh-16* expression, the negative effect from CEH-16 on *egl-18* expression, and the positive effect of EGL-18 on *ceh-16* expression (Figure 3B). These conclusions were also supported when all seam cells were included in the analysis as opposed to V1-V4 only (Figure S5). Early and late L2 stage analysis showed similar interactions between the three core genes, albeit with different strengths (Figures 3C and 3D). We found a strong repression from ELT-1 to *ceh-16* and *egl-18* at both time points. We also observed a mutual repression between CEH-16 and EGL-18. However, while in the symmetric daughters (early L2 time point), the two repressions were of approximate equal strengths, in the asymmetric daughters (late L2 time point), the repression of *egl-18* by CEH-16 was stronger than the repression of *ceh-16* by EGL-18 (Figures 3C and 3D). Taken together, while there were noticeable differences in regulatory patterns between the genes across different time points, one interaction that was consistent across time points was the repression of *egl-18* by CEH-16.

### Inferring an ordinary differential equation model explaining the gene expression data

To evaluate the validity of the *egl-18* repression by CEH-16 and how essential it is for the network in the wild type and mutant setting, we used a more mechanistic mathematical modeling approach based on ODEs. We decided to focus on the late L1 time point because in the early larval stages, it is difficult to identify a better time point when cells are as distant from cell division as in late L1 to assume a steady state. We produced an ODE-based description of the wild-type and mutant gene expression data (see STAR Methods for all the details and equations). We defined positive MRA numbers as activation actions and negative numbers as repression actions. We also included a self-activation loop for *ceh-16* as the data indicated a strong loss of *ceh-16* expression in the *ceh-16(bp323)* mutant background (note that this was absent from the MRA that is inherently incapable of detecting self-regulatory loops and that self-activation was only observed in the late L1 stage data). When multiple genes regulated another one, we assumed all the possible types of co-regulation could occur in the form of logical OR and AND actions giving rise to 20 model variants.

Our models had all possible regulatory links suggested by MRA and our data, and they included up to 11 unknown parameters. To constrain the parameters of our model variants to data, we used a recently developed Bayesian model discovery approach called SLING.^28^ SLING is a simulation-based inference method and relies on a distance measure (error) between the summary statistics of the data and the model outputs. In this case, we used the mean expression of our 3 TFs of interest in V1-V4 seam cells in the wild type and mutants at the late L1 time point as the summary statistics of the data. SLING attempts to find the simplest model that explains the data by fitting the parameters of the model, driving parameters associated with regulatory links not required to explain the data to zero. This approach helps to avoid overfitting complex models and encourages simpler models that can explain the data. In our case, SLING was particularly useful to identify interactions that are necessary in order to fit the data within a reasonable margin of error, without necessarily ruling out the actual presence of some non-essential links. Model predictions were the numerically obtained steady-state solutions of the ODE for each choice of parameters, and we used a normalized Euclidean distance between the data and model outputs (see STAR Methods for details).

**Figure 4:**
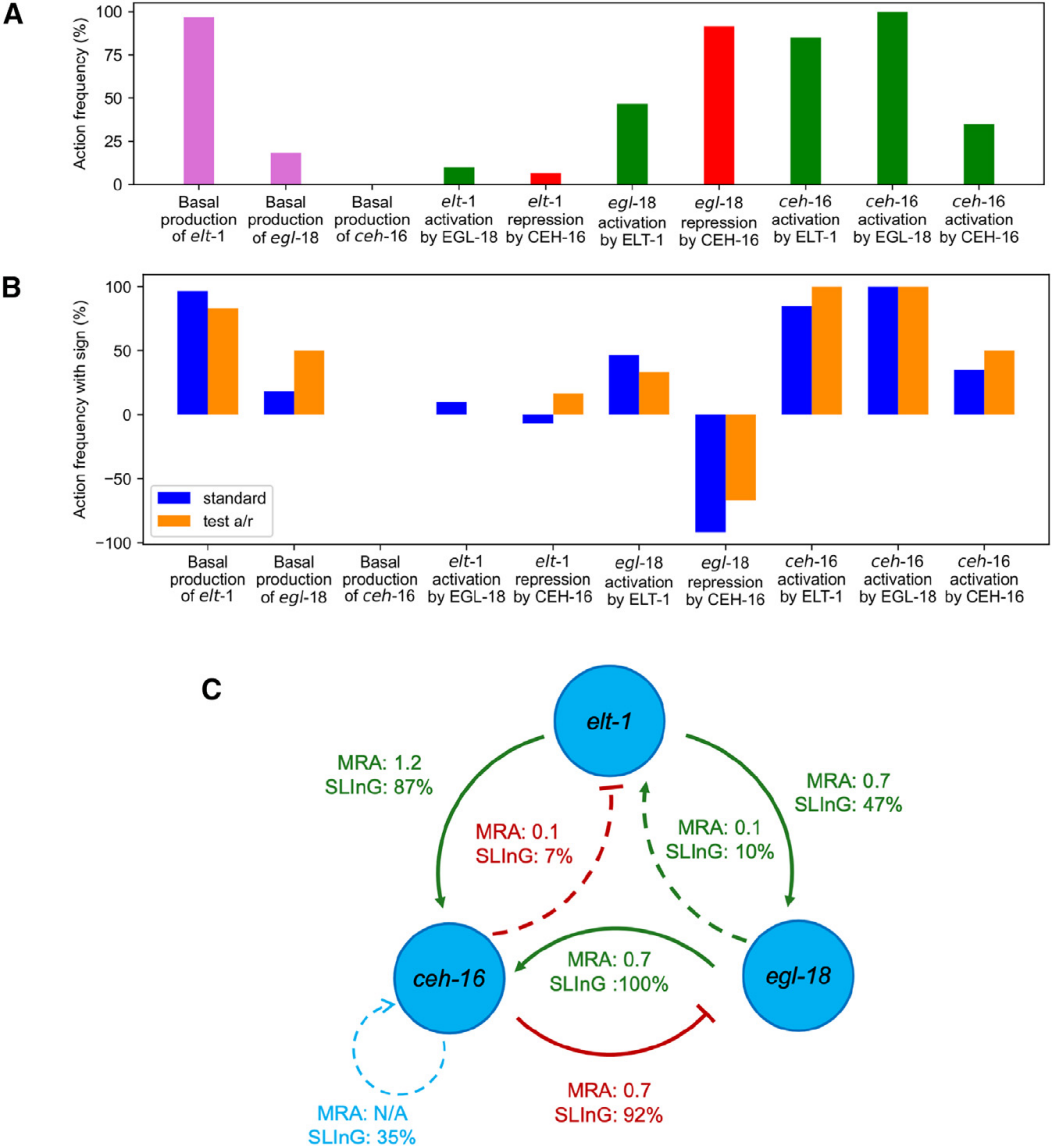
Model fitting using SLING (A) Bar graph showing the presence of each interaction in the models, computed as the percentage of all SLING runs. (B) Bar graph showing the percentage and sign of the actions of the standard model compared to the activation/repression (a/r) model. (C) GRN summarizing the MRA and SLING results. The percentages for the SLING reflect the presence of actions in final models. See also Figures S6–S10.

We performed 3 independent Markov Chain Monte Carlo runs using SLING for each of our 20 model variants. We then calculated how frequently each parameter was present in the final model fits (Figure S6A), from which we also derived the importance of links and their relationships (Figure 4A). For example, the basal production of *elt-1*, which reflects the production of *elt-1* mRNA that is independent of the levels of the other genes in the network, was highly present in the model fits in comparison to the basal production of *egl-18* and *ceh-16*. This indicates that *ceh-16* and *egl-18* expression rely on the presence of the two other core TFs while *elt-1* expression appears to be largely independent. Other highly present actions were the *egl-18* repression by CEH-16 and the *ceh-16* activation by ELT-1 and EGL-18.

We compared the final error of the 3 SLING runs of the 20 models (blue bars) to the minimal error expected from the uncertainty in the data (red dashed line) using the ABC distance measure (Figure S6B). We also compared the error of the naive model, the one with only activation actions taken from the literature, by fitting it to the data (black dashed line). All our models performed better than the naive model, with some SLING runs being as close to the data as possible given the standard deviation of the bootstrapped data (Figure S6B). This suggests that these data cannot discriminate between different modes of regulation being logical AND or OR. We also investigated the robustness of our results with regards to the level of cooperativity included and the level of sparsity desired in SLING (Figures S7 and S8, see further details in the STAR Methods).

To test specifically for the significance of the proposed repressive link from CEH-16 to *egl-18*, we decided to relax the assumption on the type of regulation and let SLING test both activation and repression (denoted here by a/r) for every single link and decide which one is best to keep or whether both can be discarded. To achieve this, we gave all regulatory parameters the option to be positive, negative, or 0. If a parameter was kept, then the action depended on its sign, and its absolute value was used as the rate. The comparison of these models with the previous SLING runs revealed a very similar behavior to the standard models tested before (Figures 4B and S6C). The *egl-18* repression by CEH-16 was one of the most conserved connections even when the sign of the parameters was relaxed. Taken together, these results reinforce the idea that CEH-16 is likely a repressor of *egl-18*.

Based on the final model fits of all model variants, we calculated the frequency in which each interaction appeared (Figure 4C). The *ceh-16* activation by EGL-18 and *egl-18* repression by CEH-16 were indeed the most robust features of the late L1 network. Both the CEH-16 and EGL-18 effect on *elt-1* was rarely present (10% and 7% respectively). The *egl-18* activation by ELT-1 was found in around half the cases (47%), and the *ceh-16* activation by ELT-1 was also highly present (87%). The *ceh-16* self-activatory loop was there in some SLING runs (35%).

The model predictions at late L1 using the median parameter set were mostly within the standard deviation of the data considered for all genes in wild type and mutants. The high mean expression of *ceh-16* in the wild type is underestimated in some model simulations, however, higher levels of *ceh-16* in wild type are obtained compared to mutants. All the calibrated models were able to achieve the right gene expression for all genes, well within the standard deviation of the data considered (Figure S9). In contrast, the comparison of the data at early L1 with the model predictions, calibrated at late L1, showed good agreement with the data for *elt-1* levels, but the levels or *egl-18* and *ceh-16* in *ceh-16*(*bp323*) and the levels of *ceh-16* in wild type were often less accurate (Figure S10).

### Model validation using double mutant expression data

**Figure 5:**
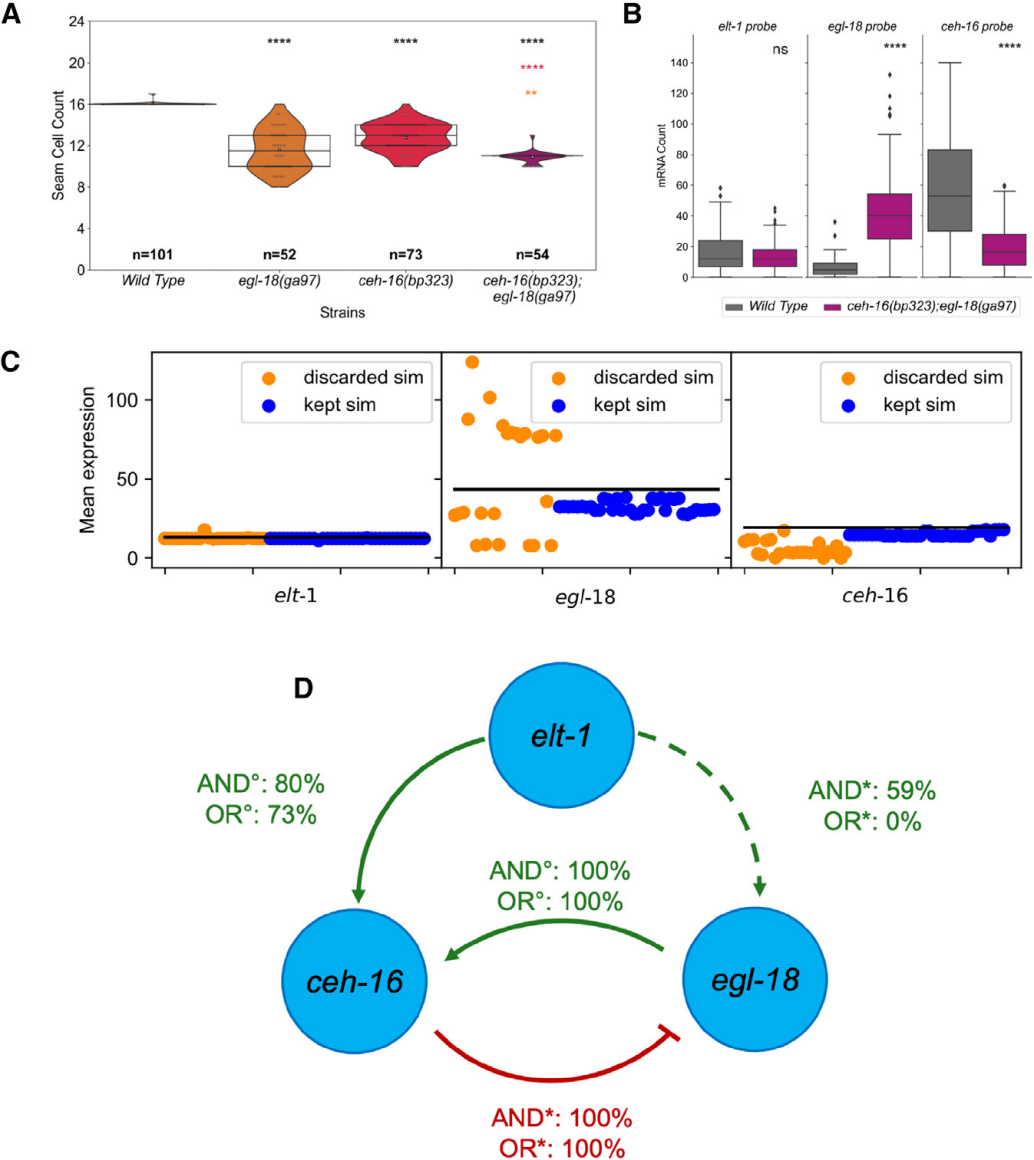
Biological testing of the model predictions in the *ceh-16(bp323);egl-18(ga97)* double mutant (A) Seam cell counts in the double *egl-18(ga97); ceh-16(bp323)* mutant in comparison to the wild type and single mutant strains at the late L4 stage. The comparison between all the mutants and wild type is denoted in black asterisk, and the comparison between the double mutant and the parental strains is denoted with asterisks in the color of the corresponding parental strain. The mean of the data is shown with a white square, the interquartile range, and the median as a boxplot. (B) Quantification of *elt-1*, *egl-18,* and *ceh-16* expression in V1-V4 seam cells via smFISH in the wild type and *egl-18(ga97); ceh-16(bp323)* double mutant at the late L1 stage; *n >* 75. The significance of the changes in expression was calculated with respect to the wild type using the Mann-Whitney U test and denoted as ∗ = *p <* 0.05, ∗∗ = *p <* 0.01, ∗∗∗ = *p <* 0.001, ∗∗∗∗ = *p <* 0.0001. The boxplots indicate the interquartile range (IQR) of the data, whiskers extend to the furthest data points within 1.5 times the IQR, black line represents the median. (C) Predicted gene expression at the late L1 stage in the double mutant from SLING runs (using the median parameter set) is compared to data (black lines). SLING runs are grouped into 2 categories based on their double-mutant error: discarded when 2.5 *<*d and kept when *d* ≤ 2.5. (D) Final model based on all kept SLING runs that are able to predict the double mutant data. The possible interacting actions on *egl-18* are indicated with the symbol ∗, while the activations on *ceh-16* are indicated with the symbol °. The percentage shown on each link is the number of times each connection is kept given an initial model that supposes an AND/OR relationship with the effect on the same gene by the other factor. The significance of the changes in A and B was calculated using the Z-test (A) and the Mann-Whitney U test (B) and denoted as ∗ = *p <* 0.05, ∗∗ = *p <* 0.01, ∗∗∗ = *p <* 0.001, ∗∗∗∗ = *p <* 0.0001. See also Figure S11.

The ODE GRN model allowed us to generate predictions as to what the behavior of the system would be upon weakening two nodes at once. Given that an unexpected outcome from the model and the data concerned the interaction between *ceh-16* and *egl-18*, we decided to construct a *ceh-16(bp323);egl-18(ga97)* double mutant. First, we compared the phenotype of the double mutant with the two parental strains by carrying out the quantification of the terminal seam cell number. We found a significant decrease in mean seam cell number and variability in the double mutant in comparison to single mutants (Figure 5A). NExt, we performed smFISH for the expression of core TFs in the double mutant background at the late L1 stage. *elt-1* mRNA levels did not change significantly from the wild type in the *ceh-16(bp323);egl-18(ga97)* background. Instead, we found that *egl-18* levels showed a significant increase compared to the wild type and that *ceh-16* levels decreased (Figure 5B).

We then tested our ODE models using these additional smFISH results. Models that performed poorly in the double mutant context (mean expressions with a distance higher than 2.5 in any of the simulations) were discarded. We found that levels of *elt-1* mRNA were accurately predicted in all models (Figure 5C). The mean expression of *egl-18* had the most variable prediction, which was particularly evident in the models we discarded. Furthermore, *ceh-16* expression was underestimated in most discarded SLING runs, indicating that the best models were the ones with the best estimate of *ceh-16* levels (Figure 5C; overall errors for the double mutant data can be seen in Figure S11A).

This analysis allowed us to delete links from the late L1 network that are discarded in 90% or more of the cases (Figure S11B), leading to the final network model for the late L1 stage (Figure 5D). It also allowed us to further characterize the OR/AND logic of some network connections. For example, the activation of *egl-18* by ELT-1 when linked with an OR logic to the repression by CEH-16 was only kept in the worst fitting SLING runs. Nevertheless, when linked with an AND logic, it was kept in 59% of the cases, indicating that this network link can only exist if *egl-18* is also repressed by CEH-16. The final network includes an incoherent feedforward loop consisting of the activation of *egl-18* and its repressor, and negative feedback between *ceh-16* and *egl-18*. Also, it further validates the existence of the negative regulation of *egl-18* by CEH-16.

## Discussion

Inference of gene regulatory networks is fundamental to increasing our mechanistic understanding of various biological processes, so there is a continuous need to develop approaches to tackle this problem at different levels. Our aim here was to use an integrative experimental and computational approach to revisit a three-factor GRN underlying seam cell development in *C. elegans*. Using smFISH analysis, we were able to quantify mRNA levels in individual seam cells in wild-type and mutant backgrounds across four developmental time points. smFISH data and *C. elegans* gene transcription can have high levels of noise, so we decided to extrapolate important interactions from this data using mathematical approaches. The adaptation of the original MRA algorithm^24^ to hypomorphic mutations has given us the flexibility to work without gene knockouts, which was necessary in this case as two out of the three core factors represent essential genes. The MRA produced an initial quantitative description of the network. Despite finding changes across different time points indicating a dynamic behavior, which is not surprising for a dividing cell population, we managed to identify a consistent interaction in early larval development, namely the repression of *egl-18* by CEH-16. In order to test whether this prediction was valid, we modeled the behavior of the network at the late L1 time point. We built on the MRA results by searching among a series of ODE models using the recently proposed Bayesian model discovery approach, SLING.^28^ This also allowed us to narrow down which interactions are essential to explain the data, resulting in several candidate ODE models using different regulatory logics. We show the CEH-16 *egl-18* repression mathematically to be highly important for the correct functioning of the network. We use the network at late L1 to generate predictions for the behavior of the network in a double mutant of *ceh-16* and *egl-18* and smFISH data obtained from the double mutant to further validate our ODE models. Taken together, this approach can both validate network links proposed by experimental analysis and predict quantitatively the strength of such interactions. We add that both the MRA approach and ODE model inference using SLInG could be extended to larger systems. While MRA is more scalable to large models than the ODE approach, the ODE inference using SLInG is more flexible for the integration of multiple types of data including time-course analysis.

Gene expression is highly stochastic due to the inherent random timing of biochemical reactions and also extrinsic variability in the cellular environment.^32^ The former type of stochasticity, which is sometimes referred to as intrinsic noise, is highly informative of the underlying regulatory pathway. Indeed, several studies have argued that this type of noise can be harnessed for learning the underlying GRN.^23^^,^^33^ However, extrinsic noise caused by global factors in the cell or tissue creates correlations between transcripts and proteins that are independent of the underlying regulatory relationship, masking correlations induced by regulation in snap-shot data.^34^ We have observed high levels of expression noise in our data, which is dominated in this case by extrinsic noise. Therefore, we decided to exclusively focus on the mean transcription expression and a deterministic (ODE) description of our GRN. In the future, methods such as dual-color smFISH,^35^ two-color TF quantification experiments^36^ or time-lapse imaging^34^ can be used to decouple extrinsic and intrinsic noise in the seam cells and investigate their link to the underlying gene regulatory architecture.

The seam cell gene network that could be derived from the literature included an activation from ELT-1 to *ceh-16* and *egl-18*, and it portrayed CEH-16 as an activator of the transcription of *egl-18*.^10^^,^^11^^,^^16^ Our smFISH and modeling data at the late L1 stage suggested that *elt-1* expression may not depend on EGL-18 and CEH-16, which is consistent with the key role for ELT-1 as a master regulator in the specification of the epidermis.^5^^,^^37^ However, we found two interesting interactions that are consistently present in our late L1 models and involve the activation of *ceh-16* by EGL-18 and the repression of *egl-18* by CEH-16. Previous work had shown *egl-18* to be regulated by CEH-16 during embryonic development; however, this interaction had been described as positive based on the observed decrease in the expression of an *egl-18p*:GFP reporter upon *ceh-16* RNAi.^10^ Instead, we find that CEH-16 negatively regulates *egl-18*, mirroring the more established repressive function of its homolog *engrailed*.^9^^,^^11^^,^^38^^,^^39^ The discrepancy between our smFISH data and the literature may reflect the different nature of the genetic perturbations used. While a *ceh-16* hypomorph leads to minimal loss of seam cell fate, strong *ceh-16* RNAi leads to seam cell fusion and high embryonic lethality, in which case loss of seam cell marker expression may not be a direct effect of CEH-16 action on *egl-18* transcription.^10^ However given that *engrailed* is known to have both repressive and activating functions,^39^ we cannot rule out that the fine-tuning of the seam cell network can vary depending on the developmental stage, as also seen in our data highlighting different interactions across time. Nevertheless, the repression of *egl-18* by CEH-16 appears to be a robust feature of the network across stages, and our modeling suggests that this interaction is essential.

Genetic analysis revealed an unexpected interaction between *ceh-16(bp323)* and *egl-18(ga97)* mutants that is compatible with the feedback loop proposed between EGL-18 and CEH-16. The double mutants showed a significant decrease in phenotypic variance in relation to the parental strains. This result was surprising because seam cell mutants tend to be generally more variable than the wild type. While the developmental basis of the restoration of developmental robustness in the double mutant background remains unknown, we hypothesize that double mutants may lose symmetric divisions at L2 in comparison to *ceh-16(bp323)* animals because the EGL-18 activation input is lacking. In addition to this, seam cell fate acquisition may be stronger in the double mutants due to the lack of repression of *egl-18* by CEH-16. Given that *egl-18(ga97)* is a nonsense mutation, the strengthening effect on seam cell fate maintenance is likely to be independent of EGL-18 and involve other factors that promote seam cell fate, with one candidate being the EGL-18 paralog ELT-6.^12^^,^^14^^,^^15^ In general, our inferred gene regulatory networks contain negative and positive feedback as well as incoherent feedforward loops. Depending on network parameters, such systems could exhibit a range of dynamic properties that, for example, explain the fast switching from symmetric to asymmetric seam cell division at the L2 stage and underlie the robust cell fate decision-making. Studies of cellular decision-making from different organisms suggest a core regulatory network structure consisting of multiple nested feedback loops, exhibiting specific nonlinear dynamical behaviors such as bi-stability or excitability required for robust cell fate determination.^40^^,^^41^^,^^42^^,^^43^ Our study adds to this body of work by revealing the architecture of the core gene-regulatory network in epidermal stem cells of *C. elegans*. Future experimental and computational characterization of the dynamical properties of this system across time could further elucidate the link to the observed cell behavior and developmental robustness.

### Limitations of the study

While the repressive interaction between CEH-16 and *egl-18* is a feature of the seam cell network that is consistent across time points, other interactions vary depending on the developmental stage and that is why we did not attempt to fit the full temporal expression of our players to the dynamics of an ODE model with fixed parameters. The observed variation in interactions could reflect additional molecular players, such as RNT-1/BRO-1, with their own dynamics that contextualize how core TFs function, as well as developmental signals that feed into this network. Furthermore, while some of the described interactions can be direct, for example, modENCODE ChIP-seq data indicate the potential direct binding of CEH-16 and ELT-1 on the *egl-18*, and *ceh-16* locus,^44^^,^^45^^,^^46^ indirect interactions are also likely to explain changes in the seam cell network topology across time. Developing tools beyond the hypormorphs utilized in our study to perturb the development of specific seam cells in a time-specific manner will be necessary in the future for validating all interactions. Future work could also expand the gene expression quantification to additional interacting partners of the three core TFs to develop a unified time-dependent model of this system as a function of developmental time. We note that our current models are deterministic and future work could focus on constructing stochastic models that could also describe the variability in gene expression across the network.

## Resource availability

### Lead contact

Further information and requests for resources and reagents should be directed to and will be fulfilled by the Lead Contact, Michalis Barkoulas (m.barkoulas@imperial.ac.uk).

### Materials availability

All unique/stable reagents generated in this study are available from the lead contact without restriction.

### Data and code availability

• Data:

Raw smFISH mRNA counts used to make the figures can be found in the GitHub repository: https://github.com/a-ceccarelli/GRN-inference.

• Code:

All code pertaining to this article is deposited in the GitHub repository: https://github.com/a-ceccarelli/GRN-inference.

• Other items:

Any additional reagent or information required to re-analyze the data reported in this article is available from the lead contact upon request.

## Acknowledgments

A.B. was supported by a 10.13039/501100000268BBSRC DTP studentship. A.C. was supported by an 10.13039/501100000266EPSRC Doctoral Training Award. A.C.S.J. was supported by the Eric and Wendy Schmidt AI in Science Postdoctoral Fellowship, a Schmidt Sciences program.

## Author contributions

A.B. and A.C. conducted the majority of the experimental and computational work. M.H. and A.C.S.J. contributed key data, code, and analysis. V.S. and M.B. supervised the work. A.B., A.C., A.C.S.J., M.H., M.B., and V.S. wrote and edited the article.

## Declaration of interests

The authors declare no competing interests.

## STAR★Methods

### Key resources table

**Table:** 

REAGENT or RESOURCE	SOURCE	IDENTIFIER
**Bacterial and virus strains**		

*E. coli* OP50	*Caenorhabditis* Genetics Center	Wormbase ID: OP50

**Chemicals, peptides, and recombinant proteins**		

PBS 10x buffer for smFISH	Ambion	AM9625
Formamide (Deionized)	Ambion	AM9342
37% Formaldehyde solution	Sigma-Aldrich	F1635-500ML
Ethanol	VWR	20821.330P
20x SSC	Ambion	AM9763
Dextran Sulfate	Sigma-Aldrich	D6001-10G
Catalase	Sigma-Aldrich	C3515-10MG
Glucose Oxidase	Sigma-Aldrich	345386-10KU
smFISH probes	Biomers	N/A

**Deposited data**		

Code, raw and analyzed data	This paper	https://github.com/a-ceccarelli/GRN-inference

**Experimental models: Organisms/strains**		

*C. elegans*: *elt-1(ku491)IV; wIs51[scm::GFP+unc-119(+)] V*	Katsanos et al.^4^	MBA650
*C. elegans*: *egl-18(ga97) IV; wIs51[scm::GFP +unc-119(+)] V*	Koneru et al.^47^	MBA290
*C. elegans*: *ceh-16(bp323ts*) III; wIs51*[scm::GFP +unc-119(+)] V*	Huang et al.^11^	HZ620
*C. elegans*: *ceh-16(bp323ts*) III; *egl-18(ga97) IV*; wIs51*[scm::GFP +unc-119(+)] V)*	This paper	MBA1216

**Oligonucleotides**		

*ceh-16(bp323)F* *5’* caggccctcgacatcgaaaa 3’	This paper	N/A
*ceh-16(bp323)R* 5’ gtgagccaattcttgtcgcc 3’	This paper	N/A
*egl-18_ga97_F* *5’* caacaccaccagcacattga 3’	This paper	N/A
*egl-18_ga97_R* *5’* tccgttgctgctaaactgtg 3’	This paper	N/A

**Software and algorithms**		

Fiji	Schindelin et al.^48^	https://imagej.net/software/fiji/
MATLAB	N/A	https://www.mathworks.com/products/matlab.html

### Experimental model and study participant details

*C. elegans* were cultured according to standard protocols.^49^ All nematodes were grown on NGM plates with *E. coli* OP50 as a food source. As reference strain, we used the JR667 strain, which contains an *scm::GFP* transgene used to visualise the seam cells (*wls51*). This transgene was introduced into the *elt-1(ku491)*, *egl-18(ga97)* and *ceh-16(bp323)* mutant backgrounds by standard crossing.

### Method details

#### Genetics

Males were induced by subjecting 50 L4 hermaphrodites to heat shock by placing them at 30°C for three hours and then 37°C for half an hour. These were screened for males 3 days later. To set up crosses, we picked males and hermaphrodites onto a plate, maintaining a ratio of 10:1 males to hermaphrodite, with at least 3 hermaphrodites on the plate to ensure a successful mating encounter. Genotyping of the *ceh-16(bp323);egl-18(ga97)* cross was based on amplification with the *egl-18_ga97_F/R* primers, as well as phenotypic observation of the egg laying defective phenotype in the mutant.

#### Single molecule Fluorescent In Situ Hybridisation (smFISH)

Synchronised animals were fixed in 4% formaldehyde in PBS at 10, 17 and 26 hours after bleaching. smFISH was performed as before^50^ using custom-made probes labelled with Cy5, listed in Table S3. Z-stacks were taken with 30 slices 0.8μm apart at 100x magnification using an oil objective using a Nikon Ti Eclipse epifluorescence inverted microscope. Exposure time was 100ms for Dapi, 3s for Cy5 and 300ms for GF5 and viewed through the NIS Nikon imaging software. The images were collected in batches of 30 animals after setting coordinates on the slide. Animals with poor probe binding or with positioning that forbade the imaging of the whole animal, were excluded.

The data analysis involved using MATLAB software to draw ROIs using the Dapi and GFP signal as markers for seam cells and to automatically quantify the mRNA spots by manually setting a brightness threshold. The thresholding was carried out independently for each worm to consider the noise levels for each image separately. The code used for spot detection is available in the GitHub repository https://github.com/a-ceccarelli/GRN-inference.

#### Building an ordinary differential equation (ODE) model from a GRN

GRNs are suitable to represent the interaction of TFs, which are proteins (denoted below in capitals, such as GENE 1), with the promoters to control transcription of mRNAs (denoted below in italics, such as *gene 1*). As transcription is typically much faster than translation, we can use time-scale separation and only write ODEs for one set of variables, assuming mRNA levels and protein levels are proportional to one another. As we have data for mRNA levels, we are assuming our variables represent mRNA levels. We describe below the detailed steps to build an ODE model to describe a GRN from its directed graph representation:

1. Create an ordinary differential equation for each *gene i* present in the network, starting by
  $$\frac{dg_{i}}{dt}=\ldots$$
  where $g_{i}$ describes the level of mRNA of *gene i* over time (t).
2. Start building the right-hand side (RHS), adding a basal term of production $b_{i}$ and subtracting a degradation term $d_{i}g_{i}$, where $d_{i}$ is a degradation rate. So, now,
  $$\frac{dg_{i}}{\text{d}t}=b_{i}+\ldots -d_{i}$$
3. Consider all the GENES that regulate *gene i.* For example, consider the case where GENE J activates *gene i* and GENE L represses it; in this scenario, we incorporate two functions to the RHS ($H_{iaj}\left(g_{j}\right)$ and ${\hat{H}}_{irl}\left(g_{l}\right)$ that represent these actions respectively (e.g. iaj is read as ‘*gene i* is activated by GENE J'). Hence, the equation becomes
  $$\frac{dg_{i}}{\text{d}t}=b_{i}+H_{\mathrm{iaj}}\left(g_{j}\right)⊕{\hat{H}}_{\mathrm{irl}}\left(g_{l}\right)-d_{i}$$
4. Substitute the symbol ⊕ with either a sum (+) or multiplication (·), depending on whether the actions of J and L in the network are related with an OR (+) or an AND (·).
5. The activation of *gene i* by the action of GENE J is described by the Hill–Langmuir function
  $$H_{iaj}\left(g_{j}\right)=v_{iaj}\frac{{g_{j}}^{\alpha_{j}}}{{k_{ja}}^{\alpha_{j}}+{g_{j}}^{\alpha_{j}}}$$
  Here, $v_{iaj}$ is the maximum activation rate of *i* by J, which is multiplied by the Hill function $\frac{{g_{j}}^{\alpha_{j}}}{{k_{ja}}^{\alpha_{j}}+{g_{j}}^{\alpha_{j}}}$. The coefficient $k_{ja}$ is the quantity of *j* needed to produce half occupation.^51^ The exponents $\alpha_{j}$ are positive numbers: when greater than 1, they represent a certain degree of cooperativity amongst genes of type J binding as activators, while when they are 1, they indicate no cooperativity.^18^
6. The repression of *gene i* by GENE L is described by the Hill–Langmuir function
  $${\hat{H}}_{irl}\left(g_{l}\right)=v_{irl}\frac{{k_{lr}}^{\alpha_{l}}}{{k_{lr}}^{\alpha_{j}}+{g_{l}}^{\alpha_{l}}}$$
  Here, $v_{irl}$ is the maximum repression rate of *i* by L, which is multiplied by the Hill function $\frac{{k_{lr}}^{\alpha_{l}}}{{k_{lr}}^{\alpha_{j}}+{g_{l}}^{\alpha_{l}}}$. The coefficient $k_{lr}$ is the quantity of *l* to produce half occupation.^51^ The exponents $\alpha_{l}$ represent the degree of cooperativity amongst genes of type L binding as repressors.^18^
7. In case a *gene i* self-activates (*i* is activated by its protein I), a function
  $$H_{iai}\left(g_{i}\right)=v_{iaj}\frac{{g_{i}}^{\alpha_{i}}}{{k_{iai}}^{\alpha_{j}}+{g_{i}}^{\alpha_{i}}}$$
  should be included. In the case of self-repression, the corresponding function ${\hat{H}}_{iri}$ should be included. The self-action term should be added to the other actions if linked with an OR or multiplied if linked with an AND.
8. Note that all parameter constants are non-negative. A zero v indicates the lack of that action. A zero k in a repression action sets the whole fraction to 0 when the denominator is non-zero, while in an activation action, it sets the fraction to 1. When two effects are multiplied (AND), their v can be substituted by one unique v.
9. The coefficients k allow the direct use of the mRNA mean expression in the equations, rather than the protein level, under the assumption that these two are proportional. Indeed, if we wanted to use the protein level $G_{i}$ instead of $g_{i}$ in the actions, where $G_{i}=\mu \;g_{i}$, then we could substitute $k_{i}$ with $K_{i}=\mu k_{i}$.
10. From this system of ODEs used to describe the wild-type data, we can obtain models to describe the mutants' data by modifying all the Hill-Langmuir functions. The activation proposed becomes
  $$H_{iaj}\left(g_{j}\right)=v_{iaj}\frac{{\left(g_{j}f_{j}\right)}^{\alpha_{j}}}{{k_{ja}}^{\alpha_{j}}+{\left(g_{j}f_{j}\right)}^{\alpha_{j}}}$$
  since the quantity of protein J is now $G_{j}=\mu g_{j}f_{j}$.

The repression becomes

$${\hat{H}}_{irl}\left(g_{l}\right)=v_{irl}\frac{{k_{lr}}^{\alpha_{l}}}{{k_{lr}}^{\alpha_{j}}+{\left(g_{l}f_{l}\right)}^{\alpha_{l}}}$$

The function $f_{j}$ (and, similarly, $f_{l}$) represents a fraction in [0,1]. The fully-working proteins J produced in the selected genotype over the quantity produced in the wild type, with the same amount of gene j mRNA. This function attains a value of 1 in the wild type since all proteins are working. Hence, their binding action is preserved, while they attain the genotype-specific value γ∈[0,1) when a mutation of gene j is considered. Note that the value of $\gamma_{i}$ can depend on the mutation's genotype.

As an example, let's now consider the following network of 3 genes: gene 1 is not influenced by the presence of gene 2 and gene 3. Gene 2 is activated by gene 1, gene 2 is repressed by gene 3, and these two actions are linked by an OR. Gene 3 is activated by gene 1 AND activated by gene 2. Gene 3 also self-activates, with an OR link with the other two activation actions of gene 3. The production of mRNA of *gene 1* is not influenced by the expression of the proteins of GENE 2 and GENE 3; hence, we can write

$$\frac{dg_{1}}{dt}=b_{1}-d_{1}g_{1}$$

We now consider the expression of *gene 2*, which is activated by GENE 1 and repressed by GENE 2. In the network, we are not specifying any link between those two actions; hence, we consider it an OR link. Thus, the equation is

$$\frac{dg_{2}}{dt}=b_{2}+v_{2a1}\frac{{\left(g_{1}f_{1}\right)}^{\alpha_{1}}}{{k_{1}}^{\alpha_{1}}+{\left(g_{1}f_{1}\right)}^{\alpha_{1}}}+v_{2r3}\frac{{k_{3}}^{\alpha_{3}}}{{k_{3}}^{\alpha_{3}}+{\left(g_{3}f_{3}\right)}^{\alpha_{3}}}-d_{2}g_{2}$$

Regarding the gene expression of *gene 3*, we have activation by GENE 1 AND GENE 2, plus a self-activation linked to them with an OR. Thus the equation is

$$\frac{dg_{3}}{dt}=b_{3}+v_{3}\frac{{\left(g_{1}f_{1}\right)}^{\alpha_{1}}}{{k_{1}}^{\alpha_{1}}+{\left(g_{1}f_{1}\right)}^{\alpha_{1}}}\cdot \frac{{\left(g_{2}f_{2}\right)}^{\alpha_{2}}}{{k_{2}}^{\alpha_{2}}+{\left(g_{2}f_{2}\right)}^{\alpha_{2}}}+v_{3s}\frac{{\left(g_{3}f_{3}\right)}^{\alpha_{3}}}{{k_{3s}}^{\alpha_{3}}+{\left(g_{3}f_{3}\right)}^{\alpha_{3}}}-d_{3}g_{3}$$

Since GENE 1 is only an activator, we identified $k_{1a2}=k_{1a3}=:k_{1}$. Moreover, we called $k_{2r3}=:k_{2}$ but kept two different k for $k_{3r2}=:k_{3}$ and $k_{3a3}=k_{3s}$ since GENE 3 is both a repressor and a self activator.

This system of 3 equations is an ODE model for the GRN. Some values can be assigned to the constants based on experimental estimates or on prior knowledge. Moreover, parameter inference algorithms can be used to find suitable parameter sets that fit the experimental data. We note that while the GRN ODE models can describe transient and temporal dynamics of gene expression in time, in this study, we are assuming that gene expression has reached a steady state, so we numerically solve for the steady-state value of expression of different genes while fitting our ODE models to the data.

For example, in the case of only OR actions, the resulting ODE model is:

(Equation 1) $$\frac{dg_{1}}{dt}=b_{1}+v_{1a2}\frac{{\left(g_{2}f_{2}\right)}^{\alpha_{2}}}{{k_{2}}^{\alpha_{2}}+{\left(g_{2}f_{2}\right)}^{\alpha_{2}}}+v_{1r3}\frac{{k_{3}}^{\alpha_{3}}}{{k_{3}}^{\alpha_{3}}+{\left(g_{3}f_{3}\right)}^{\alpha_{3}}}-d_{1}g_{1}$$

(Equation 2) $$\frac{dg_{2}}{dt}=b_{2}+v_{2a1}\frac{{\left(g_{1}f_{1}\right)}^{\alpha_{1}}}{{k_{1}}^{\alpha_{1}}+{\left(g_{1}f_{1}\right)}^{\alpha_{1}}}+v_{2r3}\frac{{k_{3}}^{\alpha_{3}}}{{k_{3}}^{\alpha_{3}}+{\left(g_{3}f_{3}\right)}^{\alpha_{3}}}-d_{2}g_{2}$$

(Equation 3) $$\frac{dg_{3}}{dt}=b_{3}+v_{3a1}\frac{{\left(g_{1}f_{1}\right)}^{\alpha_{1}}}{{k_{1}}^{\alpha_{1}}+{\left(g_{1}f_{1}\right)}^{\alpha_{1}}}+v_{3a2}\frac{{\left(g_{2}f_{2}\right)}^{\alpha_{2}}}{{k_{2}}^{\alpha_{2}}+{\left(g_{2}f_{2}\right)}^{\alpha_{2}}}+v_{3s}\frac{{\left(g_{3}f_{3}\right)}^{\alpha_{3}}}{{k_{3s}}^{\alpha_{3}}+{\left(g_{3}f_{3}\right)}^{\alpha_{3}}}-d_{3}g_{3}$$

where

$f_{1}=\gamma_{1}$ for *elt-1* mutant (*ku491*), $f_{2}=\gamma_{2}$ for *egl-18* mutant (*ga97*), $f_{3}=\gamma_{3}$ for *ceh-16* mutant (*bp323*).

Here, *elt-1* is identified as *gene 1* ($g_{1}$), *egl-18* as *gene 2* ($g_{2}$) and *ceh-16* as *gene 3* ($g_{3}$). The derivatives of their quantities in time are described by Equations 1, 2, and 3, respectively. The full list of parameters used in these equations is shown in Table S1. Degradation rates $d_{1}$, $d_{2}$ and $d_{3}$ were set to 1 since we are not modelling the time evolution of the system, and we make the assumption that the cells at the late L1 stage are at steady-state for the purposes of our ODE model. This is equivalent to scaling other kinetic rates in our model with the degradation rates. Activation and repression actions are modelled using Hill functions, as described in the STAR Methods. The functions $f_{1},f_{2},f_{3}$ represent the fraction of protein that is fully functional with 1, 2, 3 referring to ELT-1, EGL-18 and CEH-16, respectively. In ODE modelling of GRNs, co-regulation by logical OR is specified as Hill functions appearing as additive terms in the equation. In contrast, co-regulation by logical AND is specified as Hill functions appearing as multiplicative terms in the equation. We named this system of equations that assumes all actions are linked with the OR relationship (i.e. all are independent and additive) as model 1. From this model, we generated another 19 types of GRN ODE models, where the OR relationships between actions are changed to ANDs in all possible combinations, giving rise to 20 model variants described in Table S2. However, we primarily focus on models 1 and 20 for most of our analysis. We allowed the model to fit the parameters of type b, v, and k. We set $\gamma_{2}=0.01$ and $\gamma_{1}=\gamma_{3}=0.25$ to match the predicted nature of the genetic alleles with almost complete loss for EGL-18 and partial loss for ELT-1 and CEH-16 function. Moreover, we set the degradation parameters to 1, as we do not aim to fit any temporal dynamics but only the system fixed points. If degradation rates are not 1, doing this would correspond to rescaling the coefficients b and v by dividing them by the degradation rates.

To assess whether there is high cooperativity in the binding of the core TFs, we compared the model parameters trained with α=1 (no cooperativity between proteins) as opposed to α=3 (cooperativity). Here, we operate 3 SLING runs for the two extreme models, either with all actions being OR (model 1) or AND (model 20), resulting in a total of 6 *SLInG* runs. These runs revealed no significant differences when focusing on the frequency of parameters representing the network interactions (Figure S6A). The error distances of the *SLInG* runs of the two models (Figure S6B) were also very similar. While we could have also fitted the α exponents, we decided to fix α=1 in our main *SLInG* runs, as the chosen gene network does not significantly depend on the exponents of the Hill function, and thus or whether there is cooperativity or not.

Based on the double mutant analysis (Figures 5 and S8), some parameters such as the basal production of *egl-18* ($b_{2}$) or the *ceh-16* self-activation ($k_{3s}$) are only kept in models that were discarded in the context of the double mutant data, indicating that including them may result in overfitting. Therefore, the first equation (for the derivative of *elt-1*) can be described without any regulation by simply using constant synthesis and constant degradation rate:

(Equation 4) $$\frac{dg_{1}}{dt}=b_{1}-d_{1}g_{1}$$

The second equation (for the derivative of *egl-18*) can have two possible forms

(Equation 5) $$\frac{dg_{2}}{dt}=v_{2r3}\frac{{k_{3}}^{\alpha_{3}}}{{k_{3}}^{\alpha_{3}}+{\left(g_{3}f_{3}\right)}^{\alpha_{3}}}-d_{2}g_{2}$$

or

(Equation 6) $$\frac{dg_{2}}{dt}=v_{2}\frac{{\left(g_{1}f_{1}\right)}^{\alpha_{1}}}{{k_{1}}^{\alpha_{1}}+{\left(g_{1}f_{1}\right)}^{\alpha_{1}}}\cdot \frac{{k_{3}}^{\alpha_{3}}}{{k_{3}}^{\alpha_{3}}+{\left(g_{3}f_{3}\right)}^{\alpha_{3}}}-d_{2}g_{2}$$

The third equation (for the derivative of *ceh-16*) also has two possible forms

(Equation 7) $$\frac{dg_{3}}{dt}=v_{3}\frac{{\left(g_{1}f_{1}\right)}^{\alpha_{1}}}{{k_{1}}^{\alpha_{1}}+{\left(g_{1}f_{1}\right)}^{\alpha_{1}}}\cdot \frac{{\left(g_{2}f_{2}\right)}^{\alpha_{2}}}{{k_{2}}^{\alpha_{2}}+{\left(g_{2}f_{2}\right)}^{\alpha_{2}}}-d_{3}g_{3}$$

or

(Equation 8) $$\frac{dg_{3}}{dt}=v_{3a1}\frac{{\left(g_{1}f_{1}\right)}^{\alpha_{1}}}{{k_{1}}^{\alpha_{1}}+{\left(g_{1}f_{1}\right)}^{\alpha_{1}}}+v_{3a2}\frac{{\left(g_{2}f_{2}\right)}^{\alpha_{2}}}{{k_{2}}^{\alpha_{2}}+{\left(g_{2}f_{2}\right)}^{\alpha_{2}}}-d_{3}g_{3}$$

Hence, the ODE models of the final network that best describe our single-mutant and double-mutant data are obtained by combining Equations 4, with 5 or 6, and 7 or 8.

In all the *SLInG* analysis for the ODEs, we set the initial conditions of the model to be the mean values of the data points measured, as this approach leads to fast convergence to the steady state.

#### ABC distance measure

In order to fit the ODE models, we define an error distance measure d between the data for n genes and m phenotypes used and model output. This is the common practice in likelihood-free inference methods such as Approximate Bayesian Computation (ABC)^29^; hence, we refer to this as ABC distance measure or error distance measure.

We denote the mean expression of the data $\mu_{i,j}$ and their standard deviation $\sigma_{i,j}$, where i∈{1,2,…,n} indicates the gene and j∈{1,2,…,m} indicates the phenotype (wild type or mutant); both are computed using bootstrapping. We also denote the estimated fixed points as $\left(s_{1,j},s_{2,j},\ldots ,s_{n,j}\right)$, for each phenotype j as the limit of the ODE simulation and the matrix containing the approximate fixed points information with $S={\left\{s_{i,j}\right\}}_{i,j}$.

In this notation, the distance measure is defined as follows:

$$d\left(S\right)=\sqrt{\frac{1}{nm}\sum_{i=1}^{m}\frac{{\left(\mu_{i,j}-s_{i,j}\right)}^{2}}{\sigma_{i,j}}}$$

For the main inference task, we had n=3 and m=4, but in the double-mutant testing case, we only used the double-mutant data; hence, m=1.

#### Modular Response Analysis

Adapting,^24^ we implemented a Modular Response Analysis (MRA) algorithm, adding a factor that measures the change in the fully working protein production due to gene mutations. For partial-loss-of-function mutants, we have estimated the activity level at 25% due to the fact that the heterozygote behaves like a wild-type animal, and thus, the function of the gene should be below 50% but above 0%. For the complete loss-of-function mutants, we estimated the function at 0.01%.

The MRA quantifies the intensity of the influence of a module on another module. These quantifications are measured as perturbations from the steady state (wild type genetic equilibrium) to each mutant state. A module is represented by the synthesis and degradation of mRNA and the mRNA levels. The original algorithm measures the influence of the mRNA level of each gene on the synthesis of the other genes. We consider a module to be a gene's mRNA level, controlled by its synthesis and degradation, and its protein levels, which we consider to be equivalent to the mRNA mean expressions, except in mutants. Note that, for the purpose of the algorithm, assuming the same amount of protein and mRNA of a certain gene in the wild type is equivalent to assuming a proportional amount with a constant rate since the constant is simplified when considering the ratios.

The MRA algorithm produces a network interaction map (or matrix) r, whose elements estimate the type and strength of each action. Each element of the matrix $r_{ij}$ is called the local response coefficient, and it describes the effect on gene i (row) given by mutant j (column).^24^ A positive coefficient indicates an activation action, while a negative one indicates repression. The modulus of each coefficient, $\left|r_{ij}\right|$, represents the strength of the action:

• If $|r_{ij}|=1$, the relative changes in j are reproduced as equal relative changes in i.
• If $|r_{ij}|>1$, the changes in j are amplified in i.
• If $0<|r_{ij}|<1$, the changes in j are attenuated in i.
• If $|r_{ij}|=0$, j has no significant action on i.

We apply the MRA algorithm repeatedly using bootstrapping. A single iteration computes the interaction matrix, and all coefficients obtained, shown in Figure 3A. These are then averaged to compute the final interaction matrix, shown in Figure 3B.

The mRNA mean expressions (for all genes and all genotypes) are recomputed for every bootstrap sample, picking the data using random sampling with replacement.

The steps we follow to obtain the network interaction matrix (r) for each bootstrap sample are as follows (for the full mathematical derivation, see^24^). Firstly, a response matrix is computed:

$$R_{p}={\left[R_{ij}\right]}_{ij}$$

where

$$R_{ij}=2\frac{{x_{i}}^{\left(j\right)}f_{ij}-{x_{i}}^{\left(0\right)}}{{x_{i}}^{\left(j\right)}f_{ij}+{x_{i}}^{\left(0\right)}}$$

and

$f_{ij}=1$ if i≠j,

or

$f_{ij}=\gamma_{i}$ if i=j.

Here, ${x_{i}}^{\left(0\right)}$ is the mean expression of gene i in the wild type, while ${x_{i}}^{\left(j\right)}$ is its mean expression in the mutant j. The $R_{ij}$ are called central fractional differences and are computed as the finite difference of the transcript relative levels (${x_{i}}^{\left(j\right)}f_{ij}-{x_{i}}^{\left(0\right)}$) divided by their mean value $\left({x_{i}}^{\left(j\right)}f_{ij}+{x_{i}}^{\left(0\right)}\right)/2$. Subsequently, the network interaction matrix is computed:

$$r=-{\left(diag(diag\left({R_{p}}^{-1}\right)\right)}^{-1}\cdot R_{p}$$

This formula for r is based on the assumption that all $r_{ij}=-1$; thus, the presence of gene i above some level prevents a further increase of this gene expression. This corresponds to the gene's own degradation, which depends on its level. However, MRA is not able to infer other types of self-loops.

#### SLInG

We have constructed the most general ODE models based on the MRA results. We then employed *SLInG* Sparse Likelihood-free inference using Gibbs,^28^ to obtain the simplest possible submodels that can explain the data: *SLInG* is a Bayesian model-discovery tool based on a Gibbs sampling algorithm and can be used for model selection and parameter inference among a large set of related models. *SLInG* can be thought of as a sparse Approximate Bayesian Computation (ABC) method^29^ and builds on the ABC algorithm presented by Turner and Van Zandt^52^ by using sparsity-inducing priors. In Joergensen et al.,^28^ self-consistent tests based on synthetic data demonstrate that *SLInG* can reliably reverse-engineer GRNs from data and *SLInG* outperforms state-of-the-art methods that rely on constructing GRNs from expression correlation networks for an application of *SLInG* to real-world data outside of gene regulatory networks see also.^53^

*SLInG* performs sparse sampling, which encourages the algorithm to discard parameters that are not needed to explain the data. Different schemes of this type have successfully been applied for equation discovery in recent years.^25^^,^^26^^,^^36^ Within a Bayesian setting, sparsity is accomplished by including, e.g., Laplace priors for each parameter, and we take this approach in our analysis with *SLInG*.^54^^,^^55^^,^^56^ The sampling scheme of *SLInG* is hierarchical in the sense that each parameter is associated with a hyperparameter that sets the respective level of sparsity.^57^ *SLInG* autonomously calibrates these hyperparameters as it explores the parameter space. The hyperparameters are thus iteratively adjusted such that the use of superfluous parameters is highly discouraged while the algorithm is able to properly explore the parameter space along the dimensions that are necessary to describe the data. *SLInG* hence has the advantage that it can tailor the level of sparsity for each individual parameter of the model without introducing a corresponding number of free hyperparameters to be set by the user.

*SLInG* introduces an additional sparsity parameter ($\delta_{ABC}$) that sets the overall level of sparsity by scaling the distance measure, i.e. by changing the weight that the acceptance probability attributes to changes in the likelihood.^52^ For the analysis presented in this paper, we set $\delta_{ABC}$ such as to ensure a good fit to data over a high level of sparsity. For a detailed discussion on the impact and interpretation of $\delta_{ABC}$, we refer the reader to Joergensen et al., 2023.^28^

In the *SLInG* runs discussed in the results section, we thus set $\delta_{ABC}=0.0125$. In addition to the value of $\delta_{ABC}$ set by the user, *SLInG* includes a threshold for the largest possible value that the distance measure d might take for the initial guess of the parameter values. *SLInG* will autonomously readjust $\delta_{ABC}$ based on this threshold to facilitate convergence. However, after the initial burn-in phase, *SLInG* will re-evaluate the choice of $\delta_{ABC}$ and set it to the value chosen by the user if this value is consistent with the threshold for the best-fitting model within the burn-in. Here, we set the threshold to 35, i.e. *SLInG* will use $\delta_{ABC}=0.0125$ if the distance measure of the best-fitting model within the burn-in divided by 0.0125 is lower than 35.

To evaluate the impact of the choice for $\delta_{ABC}$, we repeated the analysis for a subset of ODE models using different values of $\delta_{ABC}$. We operated three *SLInG* runs for the two antipodal models, the one with all ORs (1) and the one with all ANDs (20), for four additional values of $\delta_{ABC}$, obtained by multiplying the original value of $\delta_{ABC}$ (0.0125) by 0.5, 2, 4, and 8. The *SLInG* algorithm itself automatically increases the value of $\delta_{ABC}$ if the precision for the $\delta_{ABC}$ set cannot be achieved, which happens in the first three runs for model 1 and in the second run for model 20 (Figure S7). We computed the error distance d setting the initial values of $\delta_{ABC}$ to {0.00625,0.0125,0.025,0.05,0.1} (Figure S7).

We obtained very similar errors and links when dividing or multiplying $\delta_{ABC}$ by a factor of 2. When setting $\delta_{ABC}=0.00625$, $\delta_{ABC}=0.0125$ and $\delta_{ABC}=0.025$, the final errors are thus very similar within each model, indicating that the algorithm is quite stable in a neighbourhood of the value we picked ($\delta_{ABC}=0.0125$).

However, when dividing by 2, we note that this result is partly due to the fact that the low value of $\delta_{ABC}$ comes into conflict with the aforementioned threshold. Indeed, when setting $\delta_{ABC}=0.00625$ in model 1, *SLInG* resets the value to 0.01 in all three *SLInG* runs. For model 20, on the other hand, the value is only slightly increased to 0.0075 once.

When looking at higher values of $\delta_{ABC}$ (0.05 and 0.1), the errors start to increase significantly, while the number of non-zero parameters decreases. This behaviour is to be expected: By increasing $\delta_{ABC}$, *SLInG* puts less weight on the data and thus strives towards a sparser fit, i.e. the algorithm discards more parameters at the expense of larger errors see also.^28^ For the five different values of $\delta_{ABC}$ explored in Figure S7, the average number of non-zero parameters is 7.2, 6.8, 6.8, 3.8, and 3.2, respectively, listed in the order of increasing $\delta_{ABC}$. Note that when only 3 parameters are kept in any *SLInG* run, these three parameters are identifiable as the basal productions of the three genes, i.e. no network is present. Hence, for $\delta_{ABC}=0.1$, we indeed obtained a very sparse solution in which no links are present.

### Quantification and statistical analysis

The smFISH quantifications and MRA were carried out using the code found in https://github.com/a-ceccarelli/GRN-inference.git. The p-values for comparisons of smFISH counts were calculated using Mann Whitney U test while the p-values for comparisons of seam cell quantifications were carried out using a Z-test. P-values are shown with asterisks denoted as ∗ = p *<*0.05, ∗∗ = p *<*0.01, ∗∗∗ = p *<*0.001, ∗∗∗∗ = p *<*0.0001.
