# Supplementary material for: Inference of a three-gene network underpinning epidermal stem cell development in Caenorhabditis elegans
Source: iScience. 2025 Jan 16;28(2):111826. doi: 10.1016/j.isci.2025.111826 (PMC11848479; doi:10.1016/j.isci.2025.111826)
Supplement: Document S1. Figures S1–S11 and Tables S1–S3 [file mmc1.pdf]

iScience, Volume *28*

Supplemental information

Inference of a three-gene network underpinning epidermal stem cell development in *Caenorhabditis elegans*

Alicja Brożek, Arianna Ceccarelli, Andreas Christ Sølvsten Jørgensen, Mark Hintze, Vahid Shahrezaei, and Michalis Barkoulas

	elt-1 probe				*egl-18 probe*				*ceh-16 probe*			
Wild Type	**Early L1**	**Late L1**	**Early L2**	**Late L2**	**Early L1**	**Late L1**	**Early L2**	**Late L2**	**Early L1**	**Late L1**	**Early L2**	**Late L2**
**Early L1**	0.5	9e-15	9e-75	1e-10	0.5	0.4	1e-07	0.5	0.5	2e-55	3e-05	2e-16
**Late L1**	9e-15	0.5	1e-53	8e-21	0.4	0.5	2e-05	0.4	2e-55	0.5	7e-76	1e-91
**Early L2**	9e-75	1e-53	0.5	1e-36	1e-07	2e-05	0.5	5e-05	3e-05	7e-76	0.5	6e-06
**Late L2**	1e-10	8e-21	1e-36	0.5	0.5	0.4	5e-05	0.5	2e-16	1e-91	6e-06	0.5

elt-1(ku491)	**Early L1**	**Late L1**	**Early L2**	**Late L2**	**Early L1**	**Late L1**	**Early L2**	**Late L2**	**Early L1**	**Late L1**	**Early L2**	**Late L2**
**Early L1**	0.5	0.02	1e-44	0.3	0.5	1e-15	1e-17	0.0008	0.5	5e-12	2e-35	9e-16
**Late L1**	0.02	0.5	8e-27	0.2	1e-15	0.5	1e-40	1e-19	5e-12	0.5	5e-16	0.002
**Early L2**	1e-44	8e-27	0.5	7e-24	1e-17	1e-40	0.5	4e-08	2e-35	5e-16	0.5	2e-06
**Late L2**	0.3	0.2	7e-24	0.5	0.0008	1e-19	4e-08	0.5	9e-16	0.002	2e-06	0.5

egl-18(ga97)	**Early L1**	**Late L1**	**Early L2**	**Late L2**	**Early L1**	**Late L1**	**Early L2**	**Late L2**	**Early L1**	**Late L1**	**Early L2**	**Late L2**
**Early L1**	0.5	0.3	5e-26	0.0006	0.5	6e-05	1e-50	2e-27	0.5	0.4	8e-10	0.03
**Late L1**	0.3	0.5	6e-19	0.008	6e-05	0.5	3e-23	8e-10	0.4	0.5	2e-07	0.06
**Early L2**	5e-26	6e-19	0.5	2e-22	1e-50	3e-23	0.5	1e-06	8e-10	2e-07	0.5	2e-05
**Late L2**	0.0006	0.008	2e-22	0.5	2e-27	8e-10	1e-06	0.5	0.03	0.06	2e-05	0.5

ceh-16(bp323)	**Early L1**	**Late L1**	**Early L2**	**Late L2**	**Early L1**	**Late L1**	**Early L2**	**Late L2**	**Early L1**	**Late L1**	**Early L2**	**Late L2**
**Early L1**	0.5	0.05	6e-37	0.0005	0.5	5e-15	6e-08	0.1	0.5	2e-05	0.04	0.2
**Late L1**	0.05	0.5	1e-15	0.002	5e-15	0.5	1e-05	7e-14	2e-05	0.5	0.003	3e-06
**Early L2**	6e-37	1e-15	0.5	3e-08	6e-08	1e-05	0.5	1e-06	0.04	0.003	0.5	0.02
**Late L2**	0.0005	0.002	3e-08	0.5	0.1	7e-14	1e-06	0.5	0.2	3e-06	0.02	0.5

Figure S1: A Table comparing the mRNA counts of the three core genes at different times in both wild-type and mutant backgrounds, Related to Figure 2.

In red are comparisons where the two values are significantly different, whereas in grey are values where the difference is not statistically significant (Mann-Whitney U test).

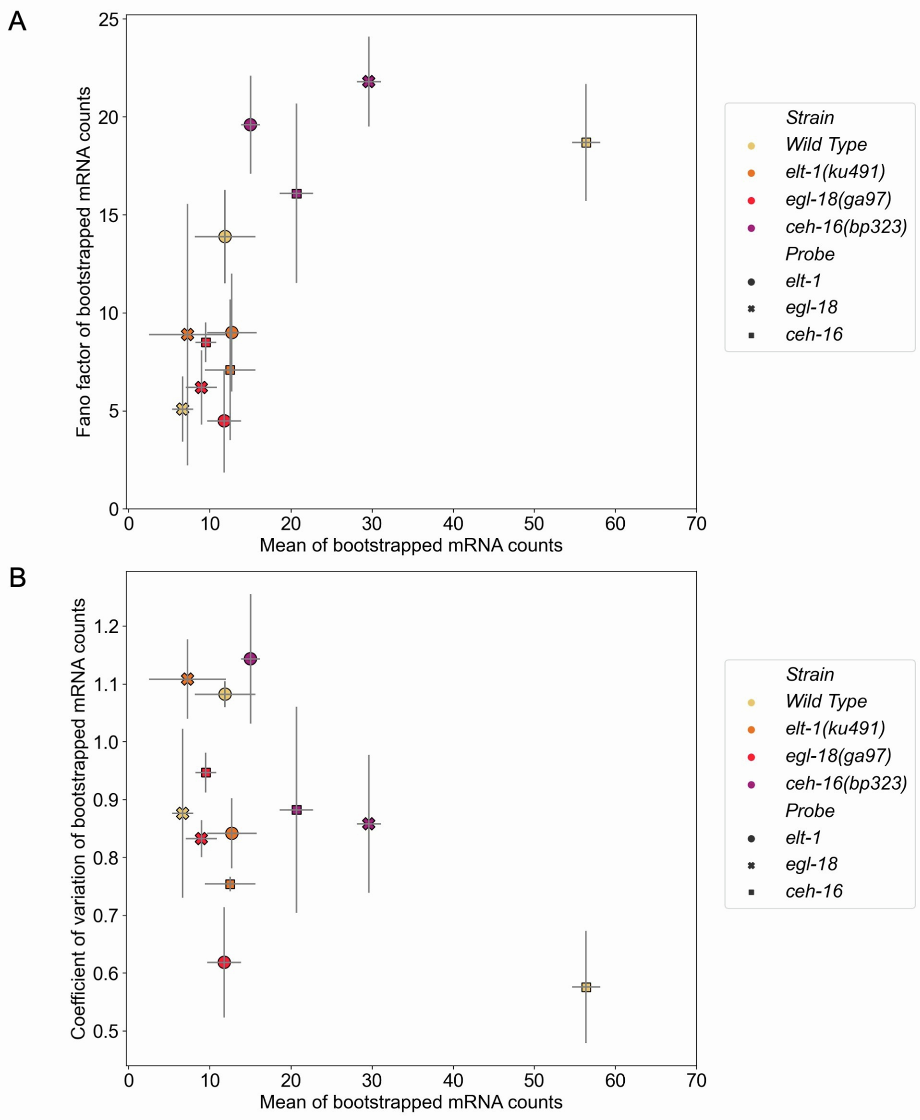

Figure S2: Noise in smFISH data of the core genes under wild type and mutant conditions, Related to Figure 2.

A: The Fano factors (calculated as $F=\frac{\sigma^2}{\mu}$) of smFISH data quantifying the variability of the core seam cell gene expression relative to the mean of each bootstrapping case. The error bars here illustrate all bootstrapping cases that fall within the interquartile range of the data. **B:** The coefficient of variation (calculated as $CV=\frac{\sigma}{\mu}$) of bootstrapped smFISH data is shown in relation to the mean. The error bars indicate the interquartile range of bootstrapped data.

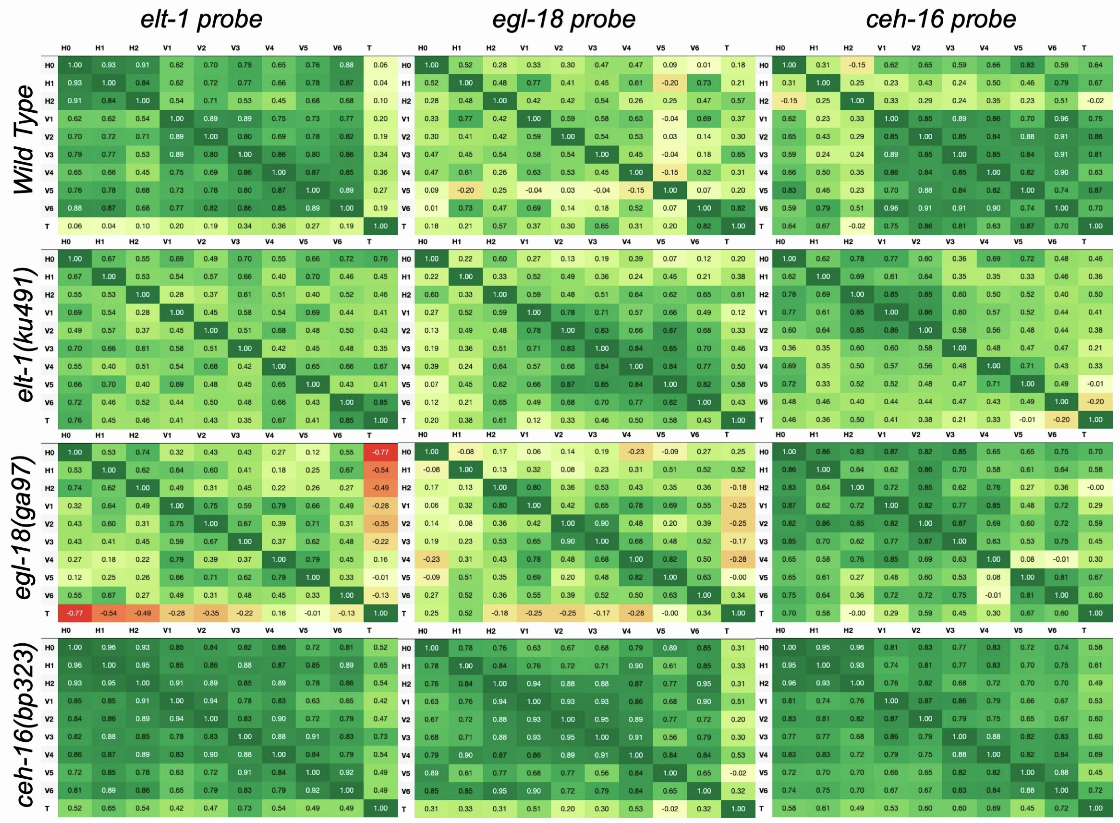

Figure S3: Correlation matrices of smFISH data of the core seam cell genes between all the seam cells under wild-type and mutant conditions, Related to Figure 2.

These matrices show the Pearson correlation coefficient of the expression of each core gene between two cells in the same animal, under mutant and wild-type conditions. Green denotes positive correlation, and red denotes negative correlation; the darker the colour, the stronger the correlation, with yellow/orange depicting weaker correlations.

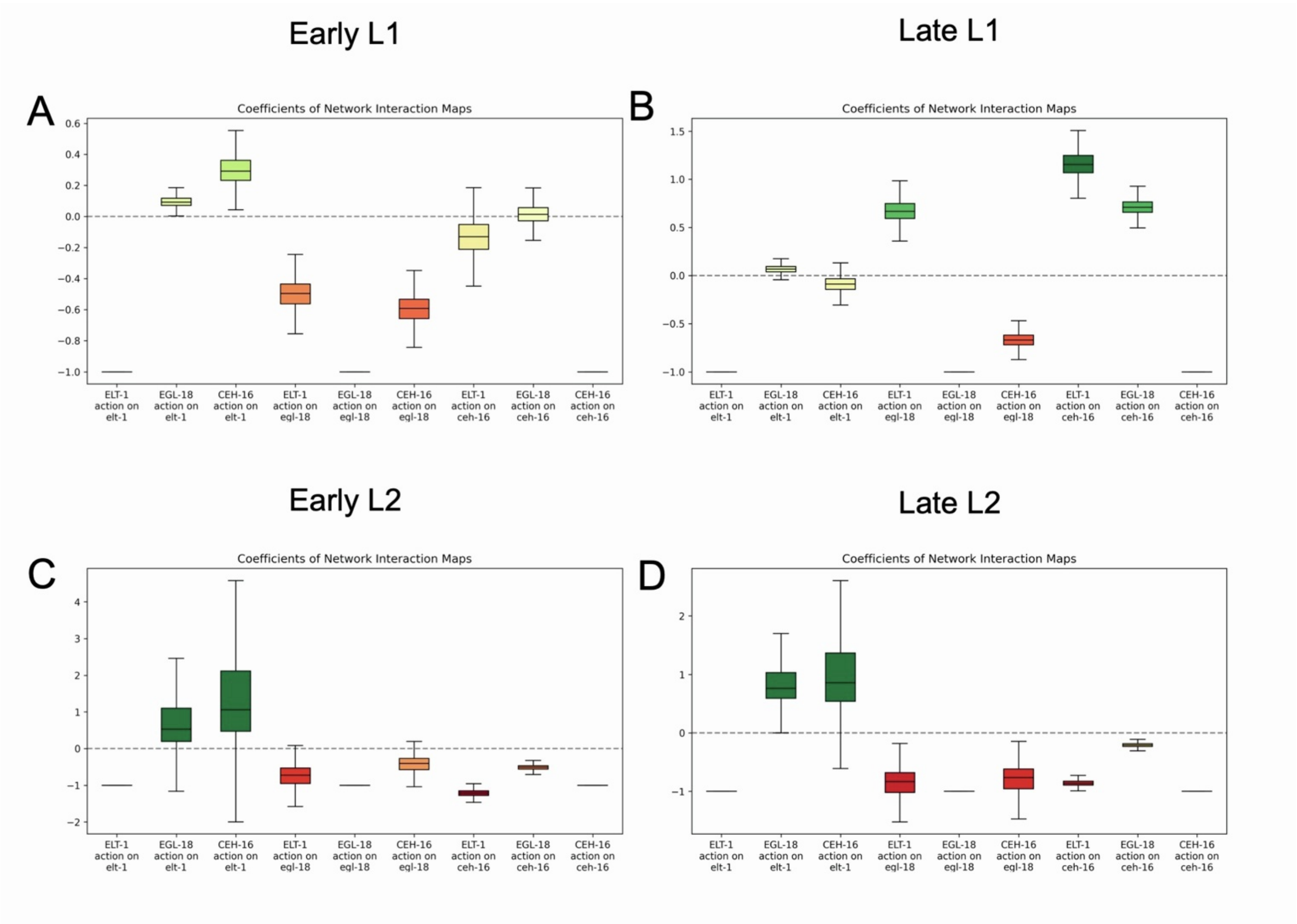

Figure S4: Interaction strength coefficients resulting from the Modular Response Analysis at all four time points, Related to Figure 3.

MRA matrix generated when V1-V4 are used at early L1 (A), late L1(B), early L2 (C)(both cell daughters of the symmetric division), and late L2 (D) (posterior cell daughters of the asymmetric division). Positive numbers correspond to activation, negative to repression, and values act as an indicator of the strength of the action. The boxplot indicates the interquartile range, and the black line in the middle is the mean.

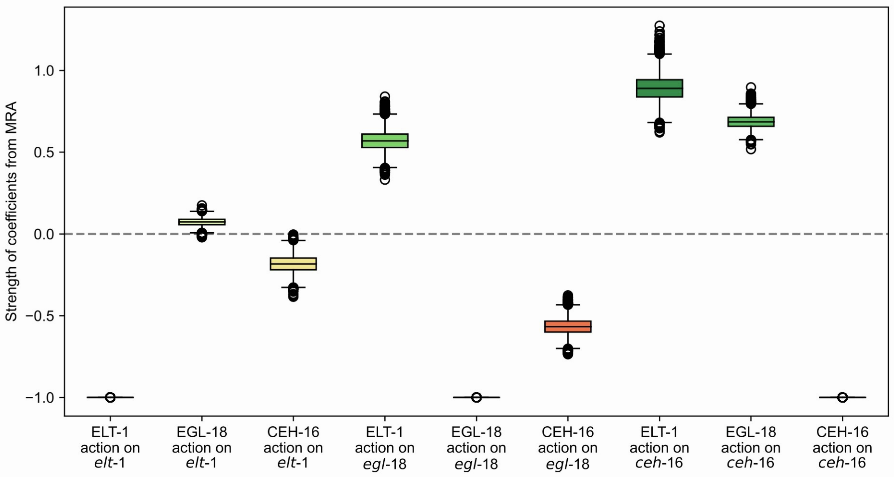

Figure S5: Interaction strength coefficients resulting from the Modular Response Analysis on all cells, Related to Figure 3.

This matrix is generated when all cells are used as opposed to V1-V4 for the late L1 stage. Positive numbers correspond to activation, negative to repression, and values act as an indicator of the strength of the action. The boxplot indicates the interquartile range, and the black line in the middle is the mean.

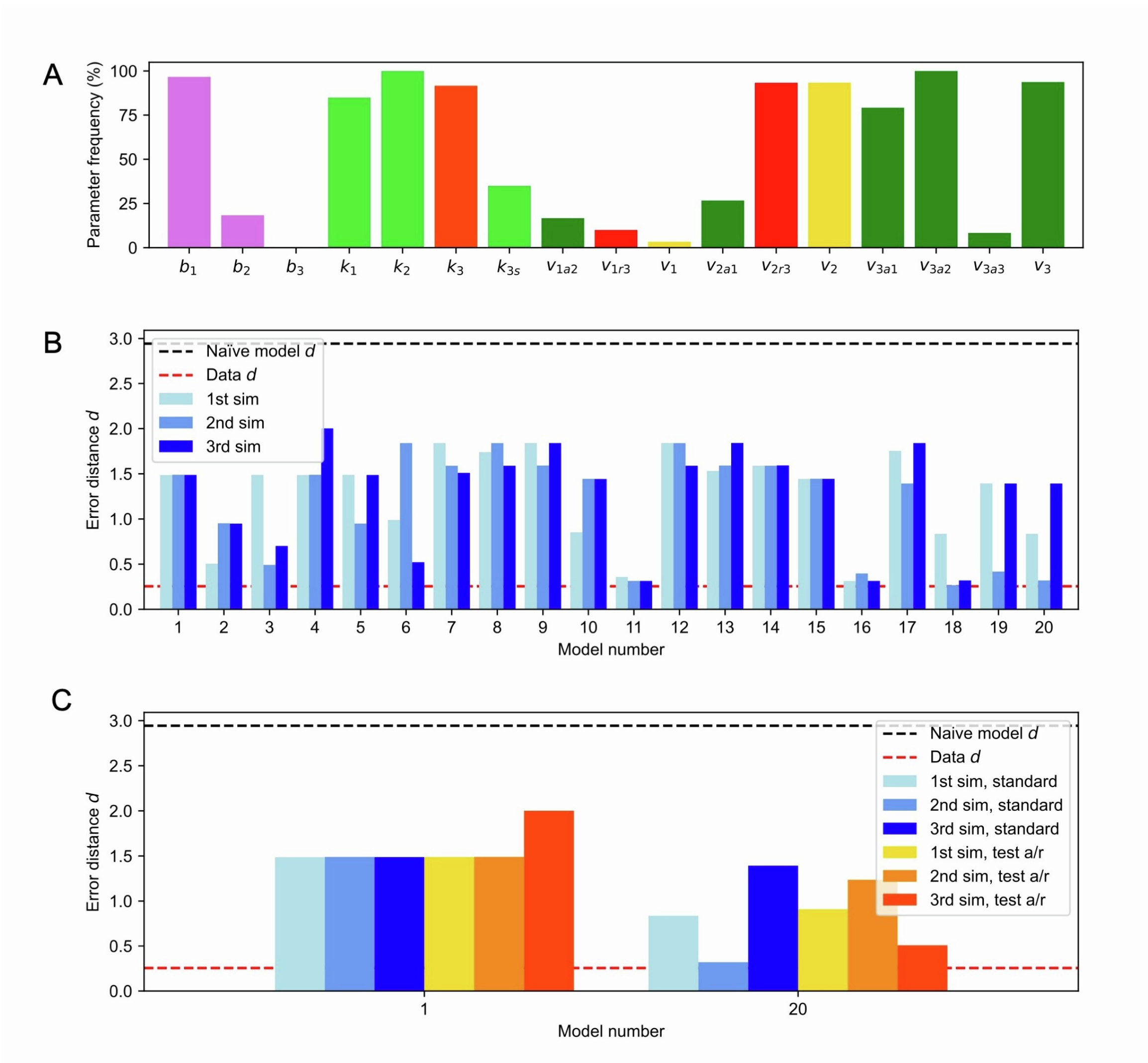

Figure S6: Testing the SLiNG model, Related to Figure 4.

A: Bar graph showing the presence of each parameter in the final models, computed as the percentage of all SLING runs. **B:** Distance measure of the median parameter set d, grouped by model type (3 SLING runs for each). The red line denoting the data value indicates one standard deviation of the original data. **C:** Distance measure of the median parameter set of the standard model compared to the activation/repression (a/r) model.

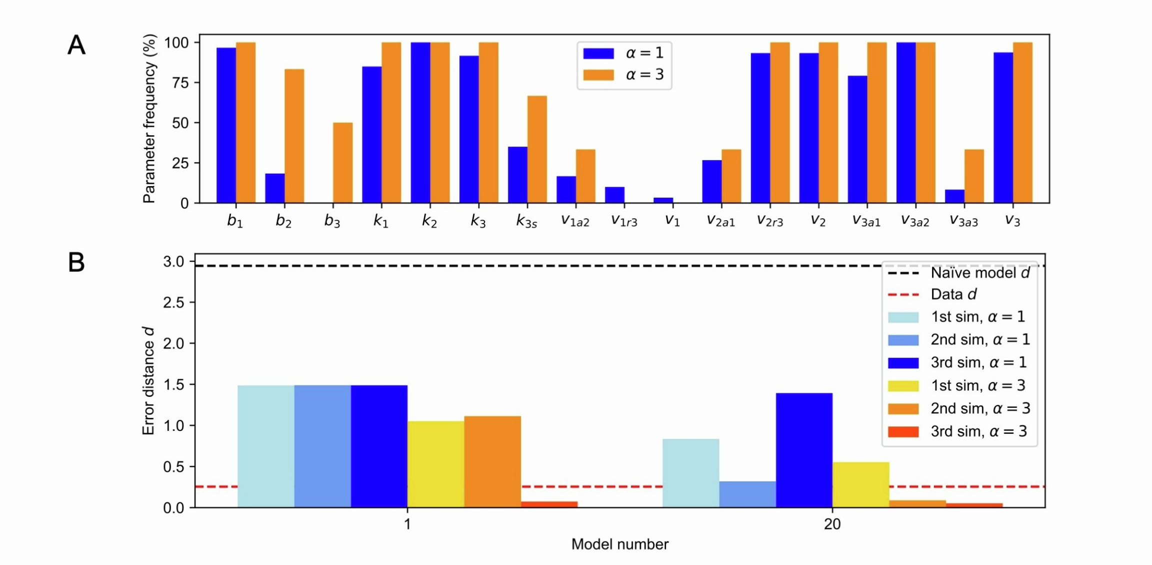

Figure S7: Testing the ODE equations with different Hill coefficients using SLiNG, Related to Figure 4.

A: Bar graph showing parameter presence in the final models comparing α = 1 (60 SLING runs, 3 for each one of the 20 models) with α = 3 (6 SLING runs, 3 for models 1 and 20). **B:** Distance measure of the median parameter set in the SLING runs for models 1 and 20 comparing α = 1 and α = 3.

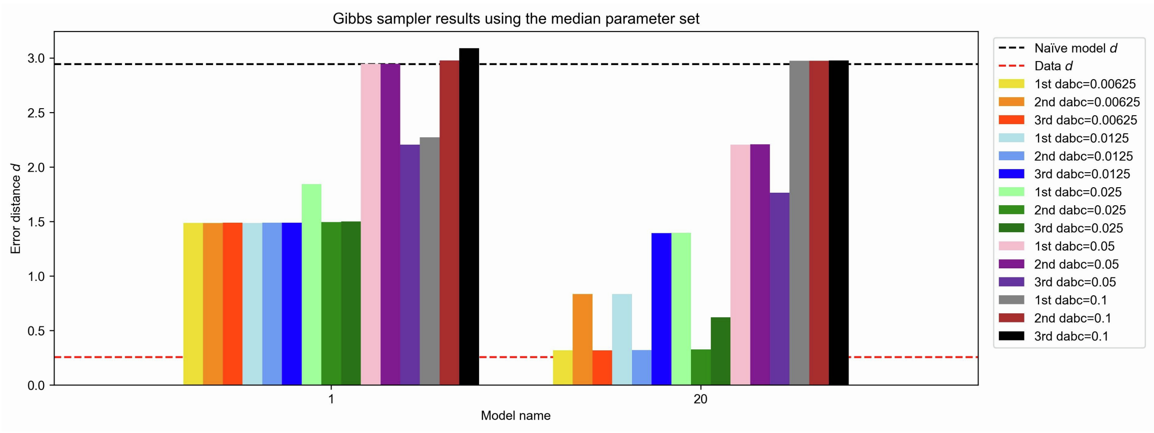

Figure S8: Testing the models with different δ_{ABC}, Related to Figure 4.

Comparison of the behaviour of the two antipodal models (1 with all OR links and 20 with all AND links) under different δ_{ABC}.

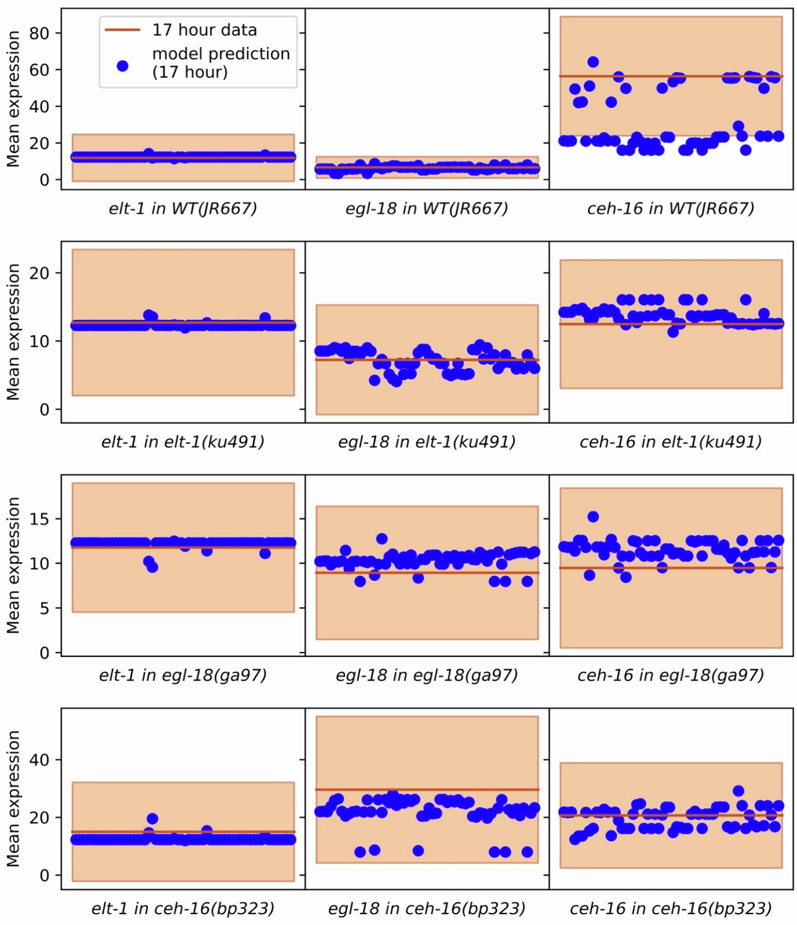

Figure S9: Comparing model predictions to data at late L1, Related to Figure 4.

Predicted gene expression using the median parameter sets from SLING runs at late L1 (blue dots correspond to simulations of the 20 models with 3 repeats) are compared to the data at the late L1 (mean expression with standard deviation for V1-V4 cells in orange).

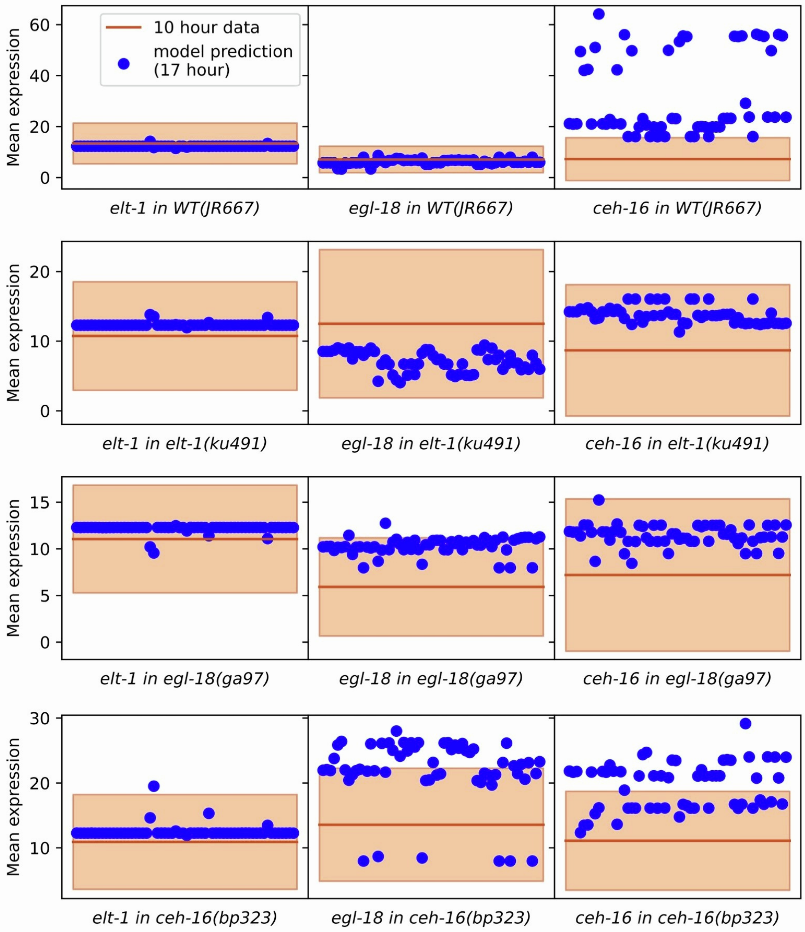

Figure S10: Comparing model predictions to data at early L1, Related to Figure 4.

Predicted gene expression using the median parameter sets from SLING runs at late L1 (blue dots correspond to simulations of the 20 models with 3 repeats) are compared to the data at the early L1 (mean expression with standard deviation for V1-V4 cells in orange).

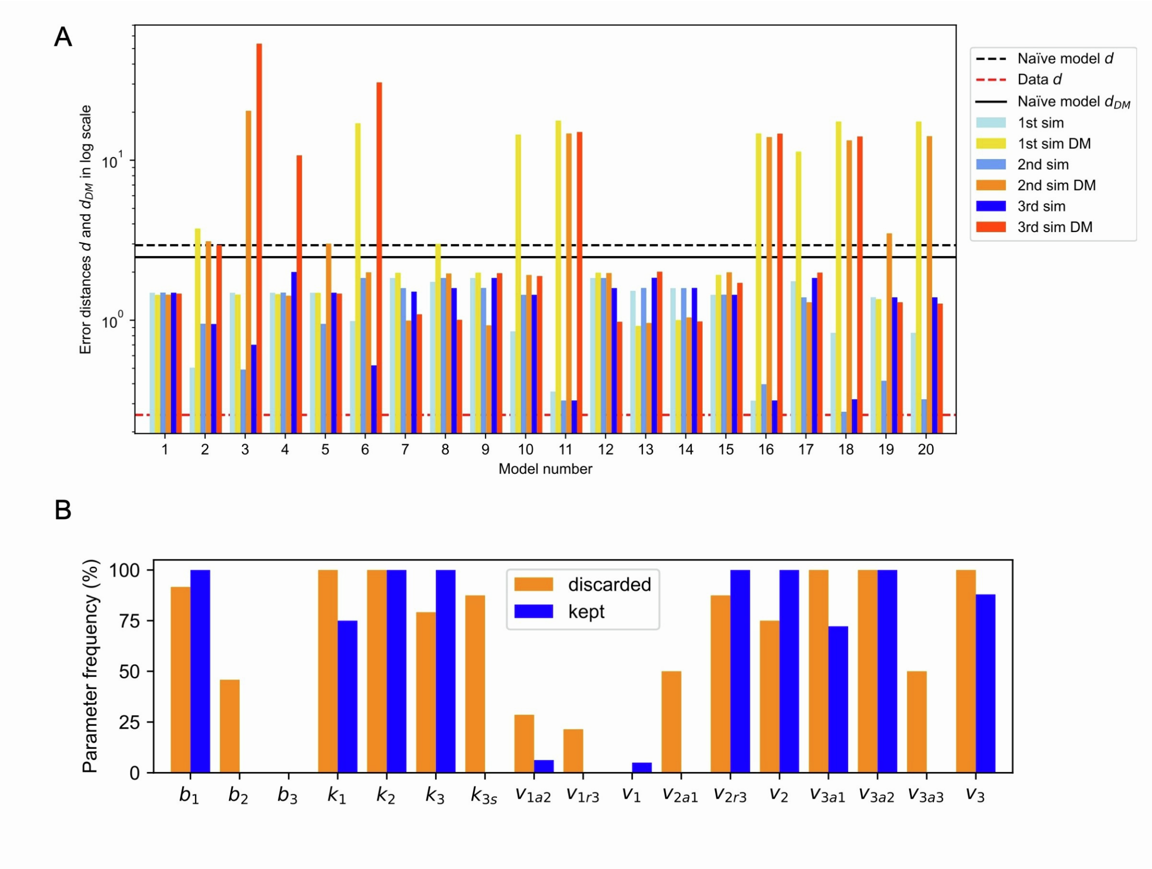

Figure S11: Testing the SLiNG model on the double mutant, Related to Figure 5.

A: Distance measure *d* (on a logarithmic scale) of the median parameter sets in predicting the wild type and single-mutant behaviour vs the double mutant behaviour. The bars are superimposed with the data presented in Figure S6B, with the lowest distance measure (depending on the SLING run) shown in front. The red line denoting the data value indicates one standard deviation of the original data. **B:** Parameter presence (in % of models) for the two categories of SLING runs is shown.

Parameter name	Parameter meaning
b_1	Basal production of *elt-1*, i.e. the amount of *elt-1* produced independently of the presence of the other two genes
b_2	Basal production of *egl-18*, i.e. the amount of *egl-18* produced independently of the presence of the other two genes
b_3	Basal production of ceh-*16*, i.e. the amount of *ceh-16* produced independently of the presence of the other two genes
k_1	The quantity of ELT-1 to produce half occupation on the activation sites, rescaled to be proportional to *elt-1* levels
k_2	The quantity of EGL-18 to produce half occupation on the activation sites, rescaled to be proportional to *egl-18* levels
k_3	The quantity of CEH-16 to produce half occupation on the repression sites, rescaled to be proportional to *ceh-16* levels
k_{3s}	The quantity of CEH-16 to produce half occupation on the activation sites, rescaled to be proportional to *ceh-16* levels
v_{1a2}	Production of *elt-1* activated by EGL-18 (OR link)
v_{1r3}	Production of *elt-1* repressed by CEH-16 (OR link)
v_1	Production of *elt-1* activated by EGL-18 AND repressed by CEH-16 (AND link)
v_{2a1}	Production of *egl-18* activated by ELT-1 (OR link)
v_{2r3}	Production of *egl-18* repressed by CEH-16 (OR link)
v_2	Production of *egl-18* activated by ELT-1 AND repressed by CEH-16 (AND link)
v_{3a1}	Production of *ceh-16* activated by ELT-1 (OR link)
v_{3a2}	Production of *ceh-16* activated by EGL-18 (OR link)
v_{3a3}	Production of *ceh-16* activated by CEH-16 (OR link)
v_3	Production of *ceh-16* activated by the AND action of 2 or 3 of the PROTEINS

Table S1: Parameter names and definition for the various ODE models used, Related to STAR methods.

	1	2	3	4	5	6	7	8	9	10	11	12	13	14	15	16	17	18	19	20
**EGL-18 activation, CEH-16 repression of *elt-1***	OR	OR	OR	AND	AND	AND	OR	OR	OR	OR	OR	AND	AND	AND	AND	AND	OR	OR	AND	AND
**ELT-1 activation, CEH-16 repression of *egl-18***	OR	OR	OR	OR	OR	OR	AND	AND	AND	OR	OR	AND	AND	AND	OR	OR	AND	AND	AND	AND
**ELT-1 activation, EGL-18 activation of *ceh-16***	OR	OR	OR	OR	OR	OR	OR	OR	OR	AND	AND	OR	OR	OR	AND	AND	AND	AND	AND	AND
**ELT-1 activation, CEH-16 activation of *ceh-16***	OR	OR	AND	OR	OR	AND	OR	OR	AND	OR	AND	OR	OR	AND	OR	AND	OR	AND	OR	AND
**EGL-18 activation, CEH-16 activation of *ceh-16***	OR	AND	OR	OR	AND	OR	OR	AND	OR	OR	AND	OR	AND	OR	OR	AND	OR	AND	OR	AND

Table S2: Description of the AND/OR logic of different models tested based on the MRA results, Related to STAR methods.

	elt-1::Cy5	egl-18::Cy5	ceh-16::Cy5
Probe Sequence	gttagcatcacgataatgca	cgtcattatgctgatcgaca	gttcaataccgaatttcaga
	agattcactttatttcggga	agcacttcgtggtgttgttg	gggtagggggaagacaggat
	ccgacaactccatctaacat	ctacacggctcatctgacgg	tgaaatagttggagatgggc
	cattcgtgttctgcatatca	cttctgtaactgtttgcaac	ttgatggacttgtagcagga
	gacggagcaaagagtccaac	tgctgattgtctttgcaaca	ggagatgcaaaagtaggaga
	gatggagtttgtgtcaggat	ttgtccattcgctccataac	cgcaatatttggtgttccat
	atgttttcctgctcaattgg	ctcgtcgagccgatactgaa	tgaacacccatgcaggatac
	aattcaacgggttttccttc	gctcattgttctctttgagc	ggtcgatcggaatatcttgt
	tccagaagaagtgccgagag	gatgagaccgatgagctttt	ctgaaaagtcgcatacctgc
	aatggtgcaatggatgcagc	cggtgatggtgaggcttttc	ctactcagagaccttctaga
	gttggttgctgatgtattgt	tctcgacaagcttcggagag	agttactcctggagtgtata
	tggataactactcggtgtgg	cctgatactggagcgactac	cacttatatccgatgtgatt
	cctccagcatattgatagtt	aagtctggaagtggactcgc	gattctcgttttcgggattt
	atgcatccatatcagttgtc	ggatcaaacatgaatccgtt	tgaacttccactacttccag
	attgacatatccattccact	gcatcattccatttggattt	tacgtggtctttttcttct
	cggattgttttgtgttccaa	ctcacggattgttgattctc	ccaattgatctccagtgaat
	gagttgtgtcgttggataga	cacggatttcgattgtggtg	cggaattcagttttgagccg
	gcgagtgtatcatatccata	gatccattggatcttcaatt	gtaatcatgattacctgctt
	cagtaattccagatgctgtc	gactcttcctgtttcacatc	tttgccgagttaggacatta
	tacgatagttccatttcctt	agtgcatccaaaaggttctg	gttgttgctggtaatcggaa
	tgttttgagtgattggctgt	gttgctgctaaactgtgctg	ccgcttttctgttagatatc
	ggtgagaggttgactgttat	tggtggaggtctggaagaac	agctcgtgagccaattcttg
	gatgatcctgaagacgtcga	gatgatgatgatggtggaga	gatctgcgattcgttgagtc
	gagtttgctgatgagcttga	ttcgatgaccgcttgtactt	gcttgttctggaaccagatt
	ggtgttcttcggtgtagaag	ttgaactgtcttggctttgg	gtcgactttttgagtttggc
	atgatcggtttgctttggag	ctcgtctcggttttctcaaa	gcatcgatctcttggaacag
	atcttccgtgctgaattgag	acggattgcagacaagcttc	tgaggatttggagtgacgga
	ctccacaattgacacactca	gtgcaatcgatagtagagcc	cctccatgaattgacggatg
	cgacgccataatggagtatt	atttctattggtcggcgaac	ccaactgagccataagttga
	atgcgttgcagaggtagttt	ttgttggatgtggtttttgc	atgtaagctcgagcctgaac
	ttcatcttgaagtagaggcc	actctttttccttgttcttt	
	ttcaccaatggacgagcatg	tggttgaagatctgtgttgc	
	tttttgagcgttctgctgtc	atcgtcggcatttgtgtgag	
	acgacagttgacacactcga	ttgaatgtgttgatggctcc	
	atttcttctccagagtgttg	ctgcgtgaattgcgagattt	
	acaagcattgcacactggat	tgctattcatgagctcttga	
	cgttccaccttatgaagttt		
	atccttcttcatggtgattg		
	ttgcgattacgggtctgaat		
	tttcttcattcttcgcgatc		
	gaagatgttgtaggcattcc		
	ccactttgatcaagttcgat		
	agtgtttttcattccccaaa		
	tgtcggtgtcatgagcattg		
	tggatgctgggaatgcatat		
	tcctcgatcgagttgaagta		
	ggcacgttttatactccaat		
	tccaaagtccaccatcattg		

Table S3: smFISH probes used in this study, Related to STAR Methods.

The sequences of the Cy5-labelled smFISH probes used to quantify *elt-1*, *egl-18* and *ceh-16* expression.
